# Unraveling adipose tissue proteomic landscapes in severe obesity: insights into metabolic complications and potential biomarkers

**DOI:** 10.1152/ajpendo.00153.2023

**Published:** 2023-10-04

**Authors:** Pavel Hruska, Jan Kucera, Daniela Kuruczova, Marek Buzga, Matej Pekar, Pavol Holeczy, David Potesil, Zbynek Zdrahal, Julie Bienertova-Vasku

**Affiliations:** ^1^Department of Pathological Physiology, Faculty of Medicine, Masaryk University, Brno, Czech Republic; ^2^Central European Institute of Technology, Masaryk University, Brno, Czech Republic; ^3^RECETOX, Faculty of Science, Masaryk University, Brno, Czech Republic; ^4^Department of Laboratory Medicine, University hospital Ostrava, Ostrava, Czech Republic; ^5^Department of Physiology and Pathophysiology, Faculty of Medicine, University of Ostrava, Ostrava, Czech Republic; ^6^Vascular and Miniinvasive Surgery Center, Hospital AGEL Trinec-Podlesi, Trinec, Czech Republic; ^7^Department of Physiology, Faculty of Medicine, Masaryk University, Brno, Czech Republic; ^8^Department of Surgery, Vitkovice Hospital, Ostrava, Czech Republic; ^9^Department of Surgical Disciplines, Faculty of Medicine, University of Ostrava, Ostrava, Czech Republic; ^10^Department of Physical Activities and Health Sciences, Faculty of Sports Studies, Masaryk University, Brno, Czech Republic

**Keywords:** obesity, proteomics, subcutaneous adipose tissue, type 2 diabetes, visceral adipose tissue

## Abstract

In this study, we aimed to comprehensively characterize the proteomic landscapes of subcutaneous adipose tissue (SAT) and visceral adipose tissue (VAT) in patients with severe obesity, to establish their associations with clinical characteristics, and to identify potential serum protein biomarkers indicative of tissue-specific alterations or metabolic states. We conducted a cross-sectional analysis of 32 patients with severe obesity (16 males and 16 females) of Central European descent who underwent bariatric surgery. Clinical parameters and body composition were assessed using dual-energy X-ray absorptiometry (DXA) and bioelectrical impedance, with 15 patients diagnosed with type 2 diabetes (T2D) and 17 with hypertension. Paired SAT and VAT samples, along with serum samples, were subjected to state-of-the-art proteomics liquid chromatography-mass spectrometry (LC-MS). Our analysis identified 7,284 proteins across SAT and VAT, with 1,249 differentially expressed proteins between the tissues and 1,206 proteins identified in serum. Correlation analyses between differential protein expression and clinical traits suggest a significant role of SAT in the pathogenesis of obesity and related metabolic complications. Specifically, the SAT proteomic profile revealed marked alterations in metabolic pathways and processes contributing to tissue fibrosis and inflammation. Although we do not establish a definitive causal relationship, it appears that VAT might respond to SAT metabolic dysfunction by potentially enhancing mitochondrial activity and expanding its capacity. However, when this adaptive response is exceeded, it could possibly contribute to insulin resistance (IR) and in some cases, it may be associated with the progression to T2D. Our findings provide critical insights into the molecular foundations of SAT and VAT in obesity and may inform the development of targeted therapeutic strategies.

**NEW & NOTEWORTHY** This study provides insights into distinct proteomic profiles of subcutaneous adipose tissue (SAT), visceral adipose tissue (VAT), and serum in patients with severe obesity and their associations with clinical traits and body composition. It underscores SAT’s crucial role in obesity development and related complications, such as insulin resistance (IR) and type 2 diabetes (T2D). Our findings emphasize the importance of understanding the SAT and VAT balance in energy homeostasis, proteostasis, and the potential role of SAT capacity in the development of metabolic disorders.

## Graphical abstract

**Figure:**
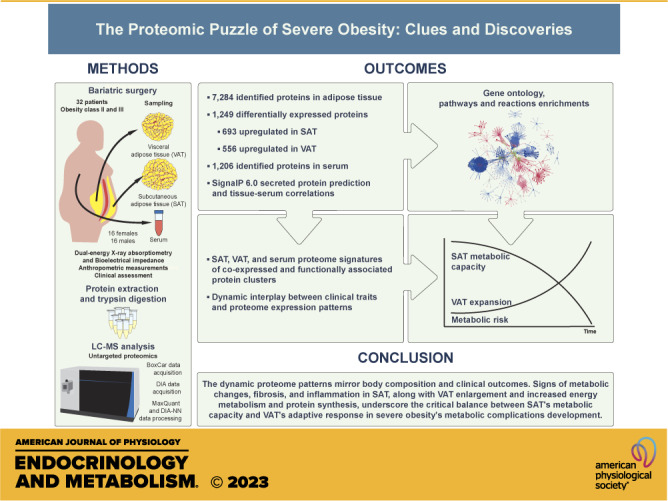

## INTRODUCTION

The worldwide prevalence of obesity and the associated risk of developing comorbidities, such as cardiovascular diseases and type 2 diabetes (T2D), has been on the rise (1). Recent studies have linked this increase in adverse health outcomes to the accumulation and dysfunction of adipose tissue (AT), particularly visceral adipose tissue (VAT) (2–5). The overall amount of VAT has been shown to be a more robust marker of cardiometabolic health risks than traditional anthropometric indicators, such as body mass index (BMI) (6). It could be suggested that the metabolic behavior of AT in various geographic locations of the body is not mutually independent. This is supported by observations of subcutaneous adipose tissue (SAT) dysfunction, which was associated with systemic inflammation and lipotoxicity, possibly contributing to VAT enlargement (7). Although both major AT depots share many properties, the functional differences between them, such as insulin sensitivity, lipolysis, adipokine secretion, and immune and inflammatory function, are crucial in the context of health outcomes, especially with increased adiposity during obesity. Despite this, the exact mechanisms through which VAT or potentially SAT contribute to disease development remain poorly understood.

Body composition is a crucial factor affecting cardiometabolic health; however, accurately determining the amount of VAT can be challenging and requires costly and time-intensive methods such as dual-energy X-ray absorptiometry (DXA), computed tomography, or magnetic resonance imaging. Consequently, having a set of comprehensive biomarkers that reflect body composition and the pathophysiological state of AT would significantly impact the management and treatment of obesity-related health issues. Recent advancements in omics technologies, particularly liquid chromatography-mass spectrometry (LC-MS), have made it possible to perform a comprehensive analysis of the molecular composition of AT, providing a deeper understanding of the complex mechanisms involved in obesity. In our previous proteomics study of the AT (8), we identified significant physiological differences between SAT and VAT adipocytes and demonstrated cell-specific expression patterns. In the present study, we focus on a description of SAT, VAT, and serum protein signatures that correlate with body composition determined by DXA and other clinical markers to assess the molecular risk patterns of obese individuals. This present study represents the most extensive characterization of the AT and serum of patients with severe obesity to date. It provides critical proteome data that shed light on AT depots’ physiological and pathophysiological roles during obesity and their contribution to its comorbidities. It also has the potential to identify complex protein biomarkers in relation to body composition, which may serve as prognostic markers.

## MATERIAL AND METHODS

### Patients

This study used paired abdominal subcutaneous and omental visceral AT biopsies from 16 females and 16 males with obesity class II and III undergoing bariatric/metabolic surgery at the Vitkovice Hospital, Ostrava, Czech Republic. Serum samples were collected presurgery, and patient selection adhered to the International Federation for the Surgery of Obesity (IFSO) guidelines. Patients did not undergo any long-term diet restrictions before surgery. All participants were white and of Central European origin. Presurgery clinical and body composition assessment included bioimpedance (BI) with the Multifrequence-Impedance-Analyzer Nutriguard-MS (Data Input, Pöcking, Germany) and DXA (Hologic, Waltham, MA) measurements. Densitometer calibration, precision error, coefficient of variation, and least significant change were established following standard procedures (9–11). Fasting blood glucose, cholesterol, triacylglycerols (TAG), high-density lipoprotein (HDL), low-density lipoprotein (LDL), and glycated hemoglobin (HbA1c) levels were also measured before surgery. Body surface area (BSA) was determined using the Du Bois formula (12). T2D was defined according to the International Diabetes Federation (IDF) criteria, with HbA1c levels ≥ 6.5% or fasting plasma glucose levels ≥ 7.0 mmol/L (13). Hypertension was defined based on the ESC/ESH guidelines, with systolic blood pressure ranging from 140 to 159 mmHg and diastolic blood pressure ranging from 90 to 99 mmHg (14). The characteristics of the patients are summarized in Table 1.

**Table 1.** Description of patients

	All Patients (*n* = 32)	Males (*n* = 16)	Females (*n* = 16)	Nondiabetic (*n* = 17)	Diabetic (*n* = 15)
Age, yr	46 ± 10	51 ± 11	42 ± 6	42 ± 8	51 ± 10
Height, cm	173 ± 10	179 ± 9	167 ± 8	175 ± 12	171 ± 8
Weight, kg	129 ± 21	137 ± 17	121 ± 23	134 ± 24	123 ± 17
Waist1, cm (inferior margin of the ribs)	124 ± 14	132 ± 11	116 ± 13	124 ± 15	124 ± 14
Waist2, cm (umbilical level)	132 ± 16	136 ± 12	127 ± 18	133 ± 18	130 ± 13
Hip, cm	133 ± 15	129 ± 14	136 ± 15	136 ± 15	129 ± 14
Waist1 to hip ratio (W1TH)	0.9 ± 0.1	1.0 ± 0.1	0.9 ± 0.1	0.9 ± 0.1	1.0 ± 0.1
Waist2 to hip ratio (W2TH)	1.0 ± 0.1	1.1 ± 0.1	0.9 ± 0.1	1.0 ± 0.1	1.0 ± 0.1
BMI, kg/m^2^	42.9 ± 5.6	43.0 ± 5.1	42.9 ± 6.2	43.6 ± 5.6	42.1 ± 5.6
BSA, m^2^	2.4 ± 0.2	2.5 ± 0.2	2.3 ± 0.2	2.4 ± 0.3	2.3 ± 0.2
Bioimpedance					
Lean body mass, kg	76 ± 17	89 ± 12	62 ± 8	76 ± 17	76 ± 17
Interstitial space, connect. tissue, L	37 ± 9	44 ± 8	30 ± 5	36 ± 10	38 ± 8
Muscle and organ cell mass, L	39 ± 10	46 ± 6	32 ± 8	40 ± 10	37 ± 10
Intracellular water, L	29 ± 7	34 ± 3	23 ± 2	28 ± 7	29 ± 7
Extracellular water, L	27 ± 6	31 ± 5	23 ± 5	28 ± 7	26 ± 6
Total water, L	56 ± 12	65 ± 9	46 ± 6	56 ± 13	55 ± 13
Body fat mass, kg	52 ± 15	48 ± 12	57 ± 17	57 ± 16	46 ± 11
Body fat proportion, %	41 ± 8	35 ± 6	47 ± 5	43 ± 7	38 ± 9
Dual-energy X-ray absorptiometry					
Lean body mass, kg	70 ± 11	79 ± 5	61 ± 8	70 ± 11	69 ± 12
Body fat mass, kg	56 ± 15	55 ± 14	57 ± 16	61 ± 17	51 ± 10
Body fat proportion, %	43 ± 6	40 ± 6	47 ± 5	45 ± 6	42 ± 6
Visceral fat mass, g	1,152 ± 353	1,274 ± 362	1,030 ± 308	1,025 ± 316	1,296 ± 348
Visceral fat volume, cm^3^	1,246 ± 382	1,378 ± 392	1,113 ± 333	1,109 ± 342	1,401 ± 376
Visceral fat area, cm^2^	239 ± 73	264 ± 75	214 ± 64	213 ± 65	269 ± 72
Android-to-gynoid ratio	1.2 ± 0.2	1.3 ± 0.1	1.1 ± 0.1	1.2 ± 0.1	1.2 ± 0.2
Clinical laboratory data					
Glycemia, mmol/L	7.1 ± 2.3	7.6 ± 2.3	6.7 ± 2.3	5.6 ± 1.1	8.9 ± 2.2
Cholesterol, mmol/L	4.8 ± 1.3	4.7 ± 1.1	4.9 ± 1.4	5.2 ± 1.2	4.4 ± 1.1
TAG, mmol/L	2.6 ± 1.7	3.0 ± 2.0	2.3 ± 1.3	2.6 ± 2.1	2.6 ± 1.2
HDL, mmol/L	1.1 ± 0.3	1.0 ± 0.3	1.2 ± 0.3	1.2 ± 0.3	0.9 ± 0.2
LDL, mmol/L	3.0 ± 1.0	3.0 ± 0.8	3.1 ± 1.1	3.3 ± 1.0	2.8 ± 0.9
HbA1c, mmol/L	5.1 ± 1.8	5.4 ± 2.0	4.9 ± 1.7	4.1 ± 0.6	6.3 ± 2.1
Hypertension, *n*	17	10	7	6	11
Type 2 diabetes, *n*	15	9	6	0	15

Average body composition and clinical characteristics of the 32 patients included in this study, were assessed before bariatric surgery. Values are means ± standard deviation. BMI, body mass index; BSA, body surface area; HbA1c, glycated hemoglobin; HDL, high-density lipoprotein; LDL, low-density lipoprotein; TAG, triacylglycerols; W1TH, waist measured at inferior margin of the ribs to hip ratio; W2TH, waist measured at umbilical level to hip ratio.

All participants signed written informed consent before participation in the study, and the design of the study was approved by the Multicentric Ethics Committee of Vitkovice Hospital, Ostrava (No. EK/3/17), in line with the principles of the Declaration of Helsinki.

### Adipose Tissue Biopsy and Serum Samples Collection

AT biopsies were collected during bariatric procedures, including laparoscopic greater curvature plication, laparoscopic sleeve gastrectomy, and Roux-en-Y gastric bypass. Surgery type had no impact on biopsy specimens. SAT biopsy was obtained from the abdominal area between the xiphoid process and the umbilicus, whereas VAT biopsy was taken from the omentum majus front part. Laparoscopic port incisions were used, following standard procedures (15, 16). AT biopsies were snap-frozen and stored at −80°C. Blood samples were collected from patients before surgery, and serum was subsequently separated using standard centrifugation procedures. The derived serum samples were then aliquoted and stored at −80°C until further analysis.

### Protein Extraction and On-Filter Digestion

Frozen AT biopsies were cut into ∼100 mg pieces and washed with ice-cold PBS. They were lysed using 400 μL of hot SDT lysis buffer [4% sodium dodecyl sulfate (SDS; Sigma-Aldrich, Cat. No. 436143), 0.1 M dithiothreitol (DTT; Thermo Fisher Scientific, Cat. No. R0862), 0.1 M Tris/HCl (Sigma-Aldrich, Cat. No. 10812846001), pH = 7.6], and incubated at 95°C for 2 h in a Thermomixer (Eppendorf). Protein extracts were processed using the filter-aided sample preparation (FASP) method using trypsin digestion (8, 17, 18). The resulting peptides were extracted into LC-MS vials (19) and concentrated in a SpeedVac concentrator (Thermo Fisher Scientific). For serum samples, 8 µL of serum was depleted of abundant proteins using High Select Top14 Abundant Protein Depletion Mini Spin Columns (Thermo Fisher Scientific, Cat. No. A36369). Depleted serum samples underwent the same FASP protocol with 90 µL of depleted sample solution.

### Proteomics Analysis

AT peptide mixtures were analyzed using an LC-MS system comprised of UltiMate 3000 RSLCnano system and Q Exactive HF-X hybrid Quadrupole-Orbitrap mass spectrometer (Thermo Fisher Scientific). Tryptic digests were concentrated and desalted on a trap column and then separated using a 90-min gradient. Mass spectrometry (MS) data were acquired using the BoxCar acquisition method (20) using MaxQuant.Live (v.1.2) (21). A supporting spectral library was created using 24 HPLC fractions of a pooled peptide mixture from subcutaneous and visceral adipocytes (8). Serum peptide mixtures were analyzed using the timsTOF Pro mass spectrometer coupled with a nanoElute HPLC system (Bruker Daltonics). The data were acquired using a 74-min gradient and data-independent acquisition (DIA).

In the analysis, MaxQuant software (v.1.6.10.43) (22) was used to process the BoxCar and spectral library MS raw data. MS ion searches were conducted against the UniProtKB Human FASTA database and a custom cRAP contaminant database. DIA serum sample data were analyzed using DIA-NN (23). The MS proteomics data and the search results have been deposited to the ProteomeXchange Consortium via the PRIDE (24) partner repository with the data set identifiers PXD041721 (AT samples) and PXD041750 (serum samples). For more details on proteomics analysis, refer to the PRIDE repository with the provided ID or Supplemental File S1 (all Supplemental Material is available at https://doi.org/10.6084/m9.figshare.22825532).

### Statistical and Bioinformatics Analyses

The MaxQuant software and DIA-NN outputs were used to process protein group (PG) data, employing a software container environment (https://github.com/OmicsWorkflows) version 4.1.3a and R software (25). For downstream analyses, the identified PGs, representing a set of proteins that share common peptide identifications, were further simplified by a selection of a representative protein, while considering their initial grouping based on shared peptides or spectral evidence. After data processing and filtering, imputed normalized protein intensities (AT proteomics data—Supplemental File S2; Serum proteomics data—Supplemental File S3) were subjected to differential expression analysis using the LIMMA R package (26). A linear model with the paired design was used to compare VAT and SAT PG intensities, with sex as a confounding variable. The LIMMA test results are provided in Supplemental File S4. The LIMMA design was also used to compare differences between males and females for SAT and VAT, respectively (Supplemental File S5), and also for serum samples (Supplemental File S6).

Gene ontology (GO) enrichment analyses were performed using ClueGO (v.2.5.8) and CluePedia plug-in (v.1.5.8) in Cytoscape 3.9.0 (27). Pathway analyses were conducted using the Reactome database (28) and String database scores (29) imported into the network visualized with ClueGo + CluePedia. Putative secreted proteins were predicted using the SignalP 6.0 Server (30) (Supplemental File S7), and correlation analysis of potentially secreted protein intensities between SAT or VAT and serum samples was performed using biweight midcorrelation (31). Finally, the relationship between SAT, VAT, serum protein expression, and clinical traits was assessed using the weighted gene coexpression network analysis (WGCNA) R package (31, 32), with modules of highly interconnected proteins and their associations with clinical traits being analyzed for functional enrichment using STRING or ClueGo + CluePedia. A more detailed description of the bioinformatics data analysis is available in Supplemental File S8.

## RESULTS

### Proteomics Results

To investigate SAT’s and VAT’s physiological and pathophysiological roles in individuals with severe obesity, we conducted an extensive untargeted proteomics study on paired SAT and VAT biopsies and serum samples from 16 male and 16 female patients with obesity class II and III. We used a label-free quantitative LC-MS approach using the BoxCar data acquisition method (20) for tissue samples and the DIA method for serum samples.

The presence of blood proteins within tissue samples significantly impacts MS analysis by affecting the dynamic range of protein concentrations. This phenomenon, attributed to blood contamination, can substantially compromise the sensitivity and accuracy of the MS analysis when investigating tissues. To address this challenge, we have adopted the BoxCar data acquisition together with the reference MS spectra library of primary SAT and VAT adipocytes peptide mixture to enhance AT proteome coverage and mitigate the effect of blood contamination. This library facilitated spectral matching in the BoxCar data, leading to the identification of 7,284 PGs, predominantly from the adipocyte proteome. We retained only proteins quantified in at least eight samples from any of the four experimental groups (SAT-female, SAT-male, VAT-female, VAT-male), yielding a final data set of 4,506 proteins for downstream analysis (Supplemental File S2).

We analyzed serum samples using the DIA method, identifying 1,206 PGs. For downstream analysis, we considered only proteins identified by proteotypic peptides and excluded proteins not quantified in at least eight female or male samples. This filtering resulted in a data set of 916 proteins (Supplemental File S3).

### Adipose Tissue Differential Protein Expression

**Figure 1.**
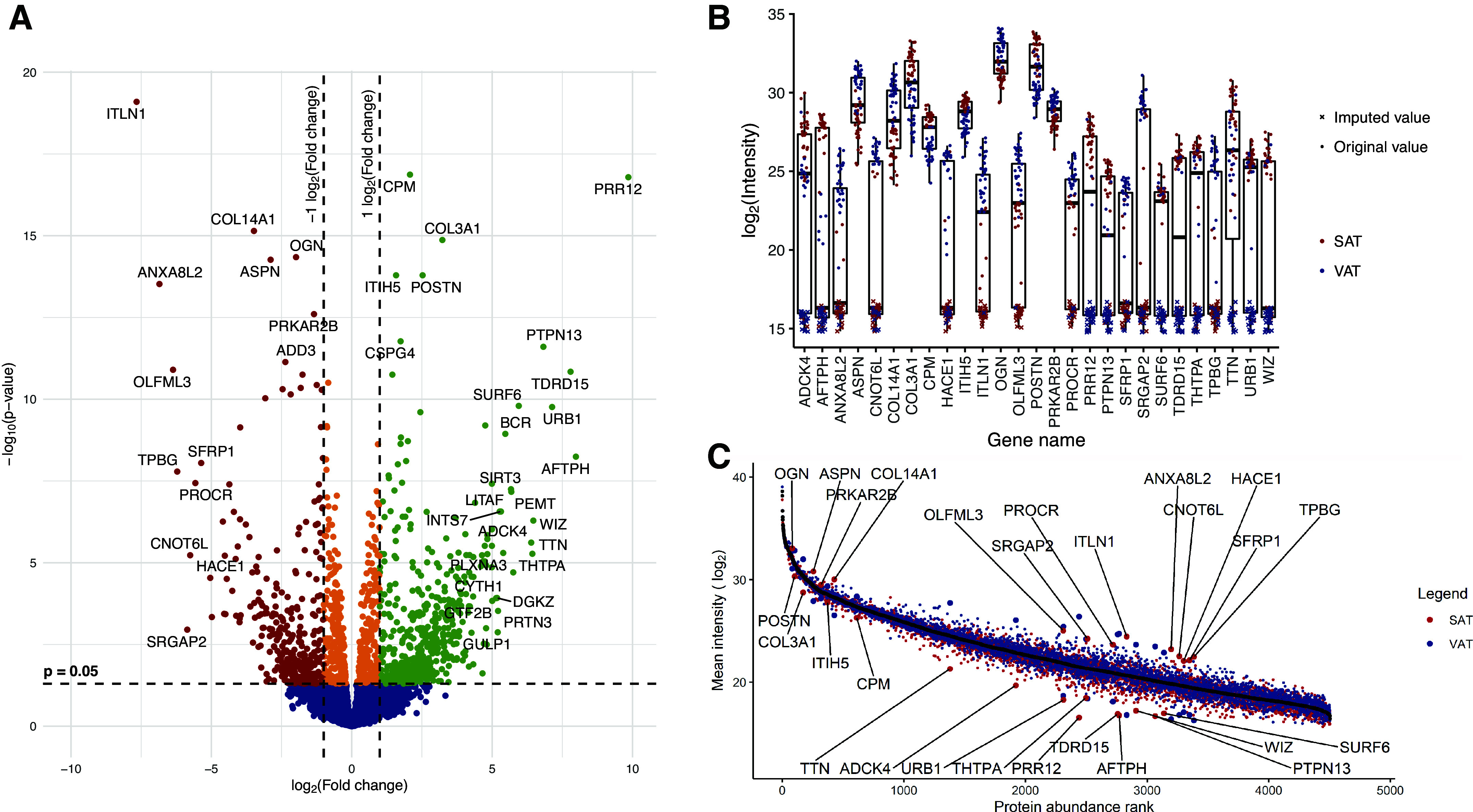
The most differentially expressed proteins in subcutaneous adipose tissue (SAT) and visceral adipose tissue (VAT). *A*: volcano plot displaying differential protein expression analysis, with significantly upregulated proteins in SAT (green) and VAT (red) highlighted. *B*: box plot illustrating the differences in intensity values between SAT and VAT for the most differentially expressed proteins, with crosses representing imputed values and dots denoting experimental intensity values. *C*: protein abundance rank plot for the most differentially expressed proteins, where the mean intensity values of all measured proteins are ordered from highest to lowest and ranked accordingly. This plot demonstrates the relative representation of these proteins within the entire adipose tissue (AT) proteome.

We performed a paired-sample LIMMA differential expression analysis, adjusted for sex, to compare protein expression in SAT and VAT. This analysis revealed 1,249 differentially expressed proteins (adj. *P* value < 0.05), with 693 upregulated in SAT and 556 upregulated in VAT. Among these, 516 proteins exhibited significant upregulation in SAT [log_2_fold change (FC) > 1], whereas 322 proteins demonstrated significant upregulation in VAT (log_2_FC > 1) (Fig. 1*A*).

Notably, the most highly upregulated proteins in SAT included proline-rich protein 12 (PRR12), aftiphilin (AFTPH), tudor domain-containing protein 15 (TDRD15), nucleolar pre-ribosomal-associated protein 1 (URB1), and tyrosine-protein phosphatase nonreceptor type 13 (PTPN13). In VAT, the most highly upregulated proteins were intelectin-1 (ITLN1), annexin A8-like protein 1 (ANXA8L1), olfactomedin-like protein 3 (OLFML3), trophoblast glycoprotein (TPBG), and SLIT-ROBO Rho GTPase-activating protein 2 (SRGAP2). The most notable changes in protein expression were observed in the middle of the detectable dynamic range (Fig. 1*C*). These log_2_FC differences between the tissues can be considered qualitative changes, primarily determined using imputed values (Fig. 1*B*).

In addition, the most significantly differentially expressed proteins, based on *P* value, were carboxypeptidase M (CPM), PRR12, collagen α-1(III) chain (COL2A1), inter-α-trypsin inhibitor heavy chain H5 (ITIH5), and periostin (POSTN) in SAT, and ITLN1, collagen α-1(XIV) chain (COL14A1), mimecan (OGN), asporin (ASPN), and annexin A8 like protein 2 (ANXA8L1) in VAT. These differences were predominantly observed in proteins with the highest expression levels in each tissue (Fig. 1*C*).

### Adipose Tissue Gene Ontology Enrichment Analysis

**Figure 2.**
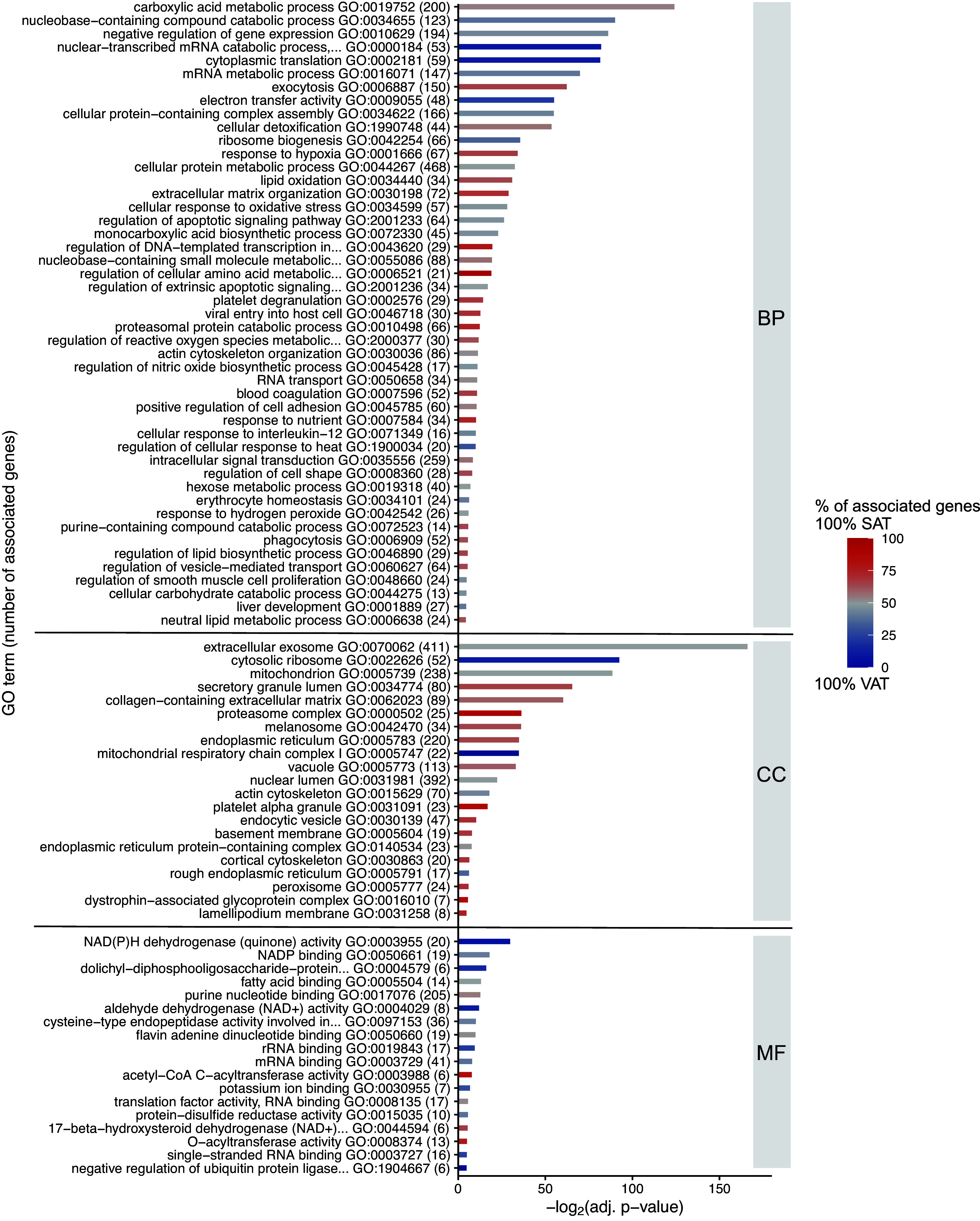
Gene ontology (GO) enrichment analysis of differentially expressed proteins in subcutaneous adipose tissue (SAT) and visceral adipose tissue (VAT). Gene ontology (GO) enrichment analysis for differentially expressed proteins in SAT (*n* = 693) and VAT (*n* = 556) conducted using Cytoscape GlueGO plug-in with a two-cluster approach. Only GO terms of levels 5–8 were considered, with a significance threshold of 0.05, and redundant groups with >50% overlap were merged. The figure displays the most significantly enriched terms (adj. *P* value <0.05) for biological processes (BP), cellular components (CC), and molecular functions (MF), emphasizing the distinct enrichments between SAT and VAT.

An in-depth analysis of differentially expressed proteins in SAT and VAT was conducted using GO. This analysis revealed significant variations in biological processes (BP), cellular component (CC), and molecular function (MF) between the two ATs (refer to Fig. 2).

Regarding BP analysis (Supplemental File S9*A*), upregulated proteins in SAT exhibited noteworthy enrichment in processes such as exocytosis, response to hypoxia, or lipid oxidation. Conversely, VAT-upregulated proteins were associated with the nucleobase-containing compound catabolic process, nuclear-transcribed mRNA catabolic process, nonsense-mediated decay, cytoplasmic translation, and electron transfer activity. Both groups of upregulated proteins were significantly enriched in the carboxylic acid metabolic process, negative regulation of gene expression, cellular protein-containing complex assembly, and cellular detoxification.

The CC analysis (Supplemental File S9*B*) highlighted that a majority of differentially expressed proteins were linked to the extracellular exosome, mitochondrion, or collagen-containing extracellular matrix (ECM). Notably, SAT-upregulated proteins were specifically associated with secretory granule lumen and proteasome complex, whereas VAT-upregulated proteins exhibited specific enrichment in cytosolic ribosome or mitochondrial respiratory chain complex I.

MF analysis (Supplemental File S9*C*) revealed NAD(P)H dehydrogenase activity as the most significantly enriched term among VAT-upregulated proteins. In contrast, acetyl-CoA C-acyltransferase activity was distinctive to SAT-upregulated proteins. Both groups of upregulated proteins displayed enrichment in NADP binding, fatty acid binding, and purine nucleotide binding.

### Adipose Tissue Pathways Enrichment Analysis

**Figure 3.**
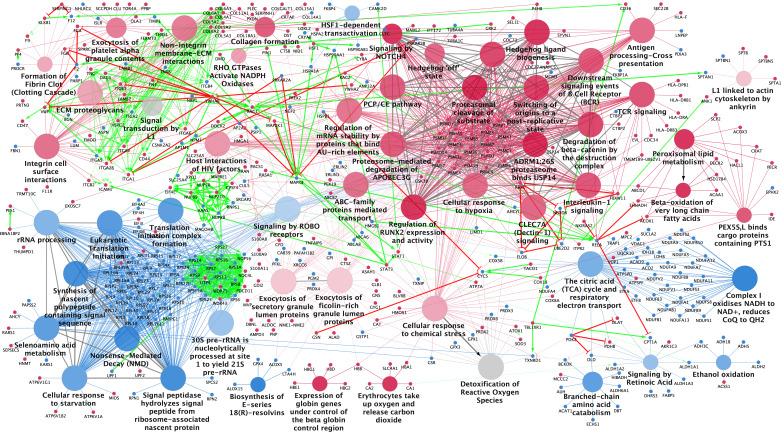
Protein pathway and interaction analysis for differentially expressed proteins in subcutaneous adipose tissue (SAT) and visceral adipose tissue (VAT). The figure presents the most significant Reactome pathways and reactions (*P* values < 0.05) for SAT and VAT differentially expressed proteins, along with associated proteins and protein-protein STRING interaction links, visualized using the Cytoscape GlueGO + CluePedia plug-in. Reactome pathway and reaction term nodes for SAT and VAT are represented as red and blue circles, respectively. Node size indicates significance, with larger nodes being more significant. Transparent nodes denote shared upregulated proteins between both groups, while gray nodes signify equal enrichment by proteins from both tissues. Bright red and green edges represent the protein’s ability to activate or inhibit the interacting protein. The most significant terms of each cluster group are displayed and manually adjusted for a reader-friendly pathway network. Comprehensive pathways and networks with group cluster affiliation can be found in Supplemental File S9.

To comprehend the disparities in signaling pathways and reactions between SAT and VAT, we engaged the Reactome database analysis tool by submitting lists of differentially expressed proteins. Subsequently, we conducted a ClueGO Reactome pathways and reactions analysis, leveraging both protein lists to underscore specific differences and the functions of related proteins (refer to Fig. 3 and Supplemental File S10).

**Figure 4.**
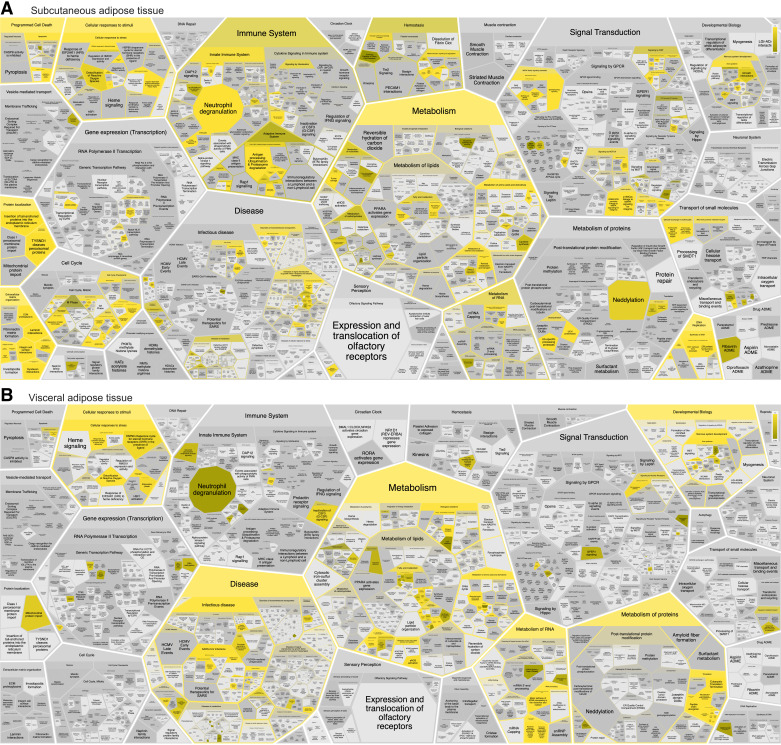
Pathway and reaction analysis of differentially expressed proteins in subcutaneous adipose tissue (SAT) and visceral adipose tissue (VAT). Reactome pathway and reactions enrichment analysis for all significantly upregulated proteins in SAT (*A*) and VAT (*B*). The figures display the most significant terms (*P* values <0.05) using the built-in Reactome Voronoi diagram visualization tool. Terms are visualized based on the Reactome event hierarchy, with the most significant terms highlighted in bright yellow.

Analyzing the Reactome event hierarchy unveiled a broader spectrum of diverse pathways among SAT-upregulated proteins compared with VAT-upregulated proteins (Fig. 4 and Supplemental File S11, *A* and *B*). Among SAT-upregulated proteins, the most significantly enriched root terms encompass ECM organization, programmed cell death, and metabolism. Conversely, among VAT-upregulated proteins, the most significantly enriched term was metabolism, followed by a cellular response to stimuli and the metabolism of RNA.

The distribution of SAT-upregulated proteins across various pathways underscores the distinct characteristics of AT depots. Our findings reveal that although both SAT- and VAT-upregulated proteins are implicated in stress response, SAT-upregulated proteins are predominantly engaged in cellular responses to hypoxia and chemical stress. In contrast, VAT-upregulated proteins are linked to cellular responses to starvation. This distinction is further highlighted by several proteins involved in signal transduction, including Hedgehog, NOTCH4, and the MAPK signaling family members, all associated with proteasome proteins. An association with diseases related to signal transduction by growth factor receptors and second messengers was also observed.

Our results also reveal substantial associations with ECM organization in SAT, including proteins engaged in collagen formation, laminin interactions, nonintegrin membrane-ECM interactions, ECM proteoglycans, and ECM degradation, along with integrin cell surface interactions. This highlights the distinct structural and functional attributes of SAT in relation to ECM dynamics. Furthermore, the regulation of the mitotic cell cycle, encompassing proteins governing cell cycle checkpoints, apoptosis, DNA replication, DNA synthesis, and mRNA stability via AU-rich element binding proteins, further reinforces the unique nature of SAT.

Numerous immune system pathways also exhibited enrichment with SAT-upregulated proteins, including neutrophil degranulation, T cell receptor signaling, B cell receptor signaling, and the TNFR2 noncanonical NF-kB pathway. Furthermore, the ABC-family protein-mediated transport pathway was also enriched in SAT, potentially involving proteins associated with disorders of transmembrane transporters. In contrast, VAT-upregulated proteins predominantly enriched the citric acid cycle and respiratory electron transport, along with proteins involved in RNA metabolism, including rRNA processing, nonsense-mediated decay, and eukaryotic translation.

### Adipose Tissue Protein Secretion and Serum Protein Correlation

To understand the link between AT and serum protein levels, we conducted a biweight midcorrelation analysis of all proteins detected in both SAT and VAT, as well as serum samples (Supplemental File S12). Initially, among the overlapping proteins (*n* = 554), we found 55 in SAT and 60 in VAT that significantly correlated with the serum protein levels. However, a notable subset of these proteins was associated with blood-borne components, like immunoglobulins and coagulation cascade proteins, present due to the blood content in the tissues. To pinpoint proteins likely moving from tissues to serum, we used SignalP-6.0 to predict potentially secreted proteins and further focus on those with secretion potential or differential expression between SAT and VAT.

ERAP1, a potentially secreted protein, demonstrated the most significant positive correlation with both SAT and VAT (cor_SAT_ = 0.75, cor_VAT_ = 0.76). Adiponectin, significantly upregulated in SAT and possibly secreted, displayed a significant correlation between its SAT tissue expression and serum levels (cor_SAT_ = 0.61). Furthermore, several other potentially secreted SAT-upregulated proteins, such as semaphorin-3-C (SEMA3C) (cor_SAT_ = 0.52), apolipoprotein A-IV (APOA4) (cor_SAT_ = 0.52), extracellular superoxide dismutase [Cu-Zn] (SOD3) (cor_SAT_ = 0.43), apolipoprotein D (APOD) (cor_SAT_ = 0.39), and apolipoprotein C-III (APOC3) (cor_SAT_ = 0.36), exhibited significant correlations with SAT expression levels in the serum. While apolipoprotein M (APOM), not classified as a potentially secreted protein but upregulated in SAT, showed a significant correlation with both SAT and VAT expression levels (cor_SAT_ = 0.46, cor_VAT_ = 0.49).

The most substantial serum correlation exclusively with VAT protein intensities was seen with coagulation factor XII (cor_VAT_ = 0.58), although its presence in the tissue is likely due to blood contamination. Serum amyloid A-4 protein (SAA4) positively correlated with VAT protein levels (cor_VAT_ = 0.49), despite lacking differential expression between SAT and VAT. Notably, a negative correlation was observed between nidogen-1 (NID1) serum levels and VAT levels (cor_VAT_ = −0.45); although NID1 is a potentially secreted protein, its tissue expression was higher in SAT compared with VAT.

Finally, a positive correlation emerged between serum and VAT levels for 6-phosphogluconate dehydrogenase (decarboxylating) (PGD) (cor_VAT_ = 0.40), which is slightly upregulated in VAT. Leptin (cor_VAT_ = 0.39) and apolipoprotein B-100 (APOB) (cor_VAT_ = 0.38), both potentially secreted proteins, also demonstrated positive correlations between serum and VAT protein levels while concurrently exhibiting cross-correlation with SAT intensities (cor_SAT-VAT_ = 0.57 and cor_SAT-VAT_ = 0.50, respectively).

### Weighted Gene Coexpression Network Analysis

**Figure 5.**
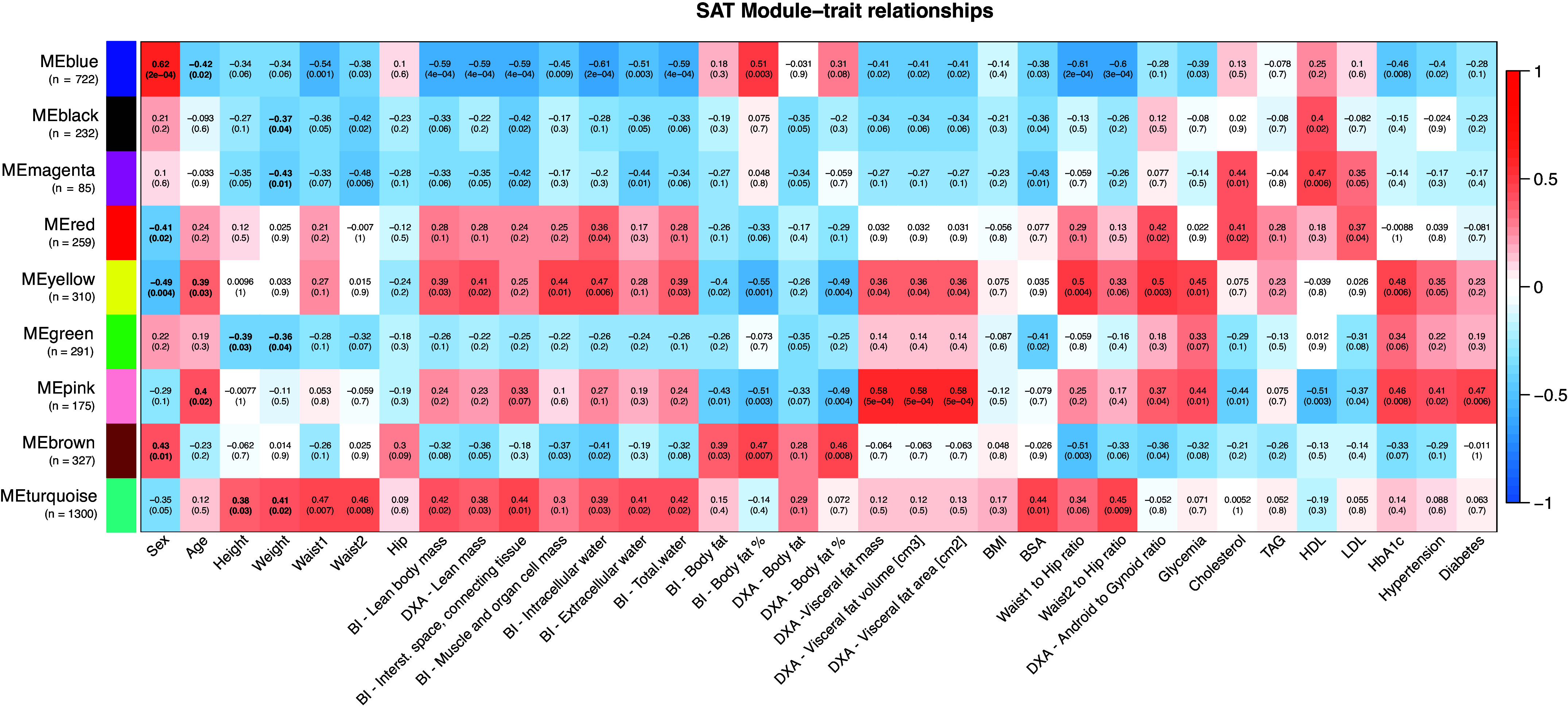
Subcutaneous adipose tissue (SAT) module-traits relationships. Relationships between SAT module eigengenes derived from weighted gene coexpression network analysis (WGCNA) and selected clinical variables, along with corresponding Pearson correlation *P* values. Strong positive correlations are represented in red, while strong negative correlations are in blue; numbers within individual cells indicate the correlation coefficient and (*P* value). Significant module-trait correlations (*P* value < 0.05) with an absolute correlation value >0.5 are highlighted in bold. Module sizes are provided in brackets. Waist1 refers to waist circumference measured at the inferior margin of the ribs, and Waist2 denotes measurements at the umbilical level. BI, bioimpedance; BMI, body mass index; BSA, body surface area; DXA, dual-energy X-ray absorptiometry; HDL, high-density lipoprotein; LDL, low-density lipoprotein; TAG, triacylglycerols.

**Figure 6.**
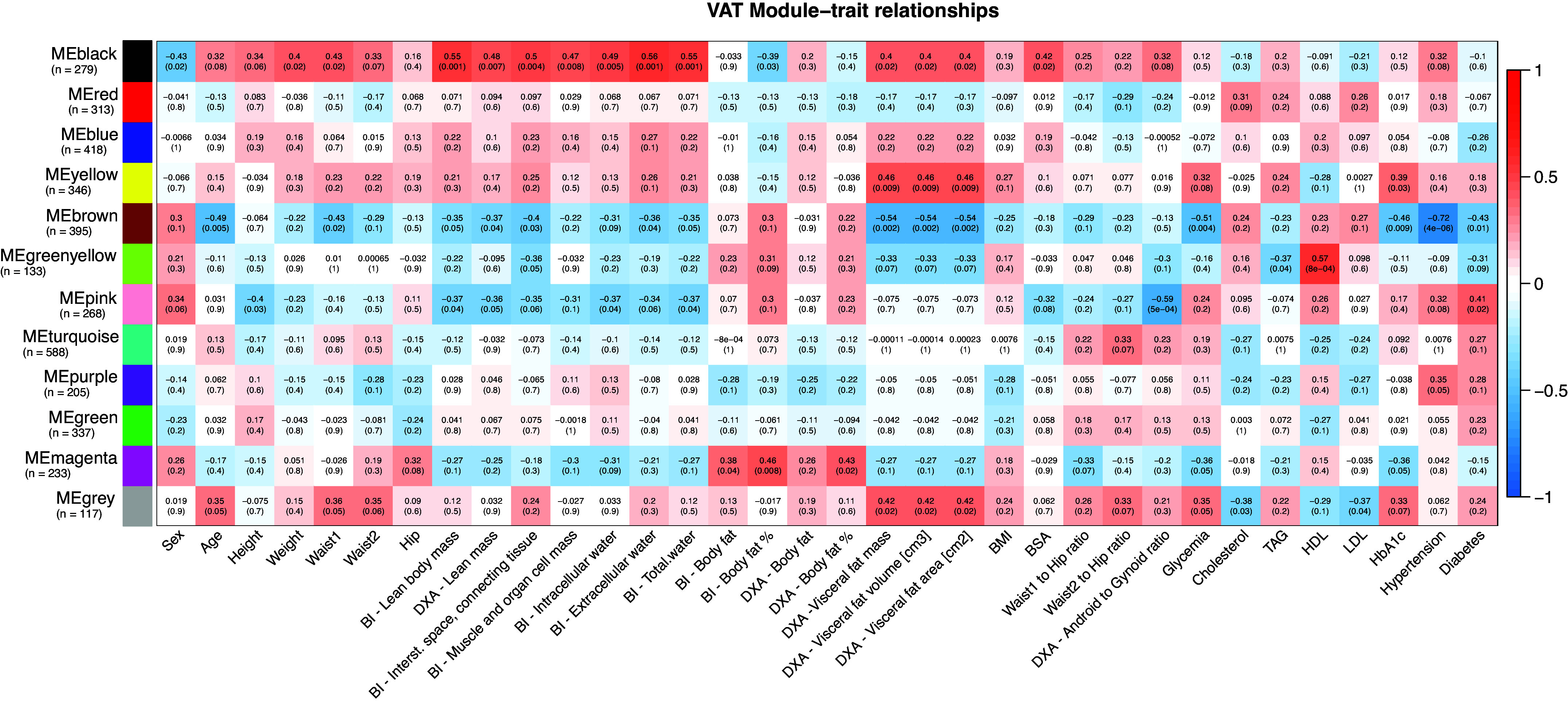
Visceral adipose tissue (VAT) module-traits relationships. Relationships between VAT module eigengenes derived from weighted gene coexpression network analysis (WGCNA) and selected clinical variables, along with corresponding Pearson correlation *P* values. Strong positive correlations are represented in red, while strong negative correlations are in blue; numbers within individual cells indicate the correlation coefficient and (*P* value). Significant module-trait correlations (*P* value < 0.05) with an absolute correlation value >0.5 are highlighted in bold. Module sizes are provided in brackets. Waist1 refers to waist circumference measured at the inferior margin of the ribs, and Waist2 denotes measurements at the umbilical level. BI, bioimpedance; BMI, body mass index; BSA, body surface area; DXA, dual-energy X-ray absorptiometry; HDL, high-density lipoprotein; LDL, low-density lipoprotein; TAG, triacylglycerols.

**Figure 7.**
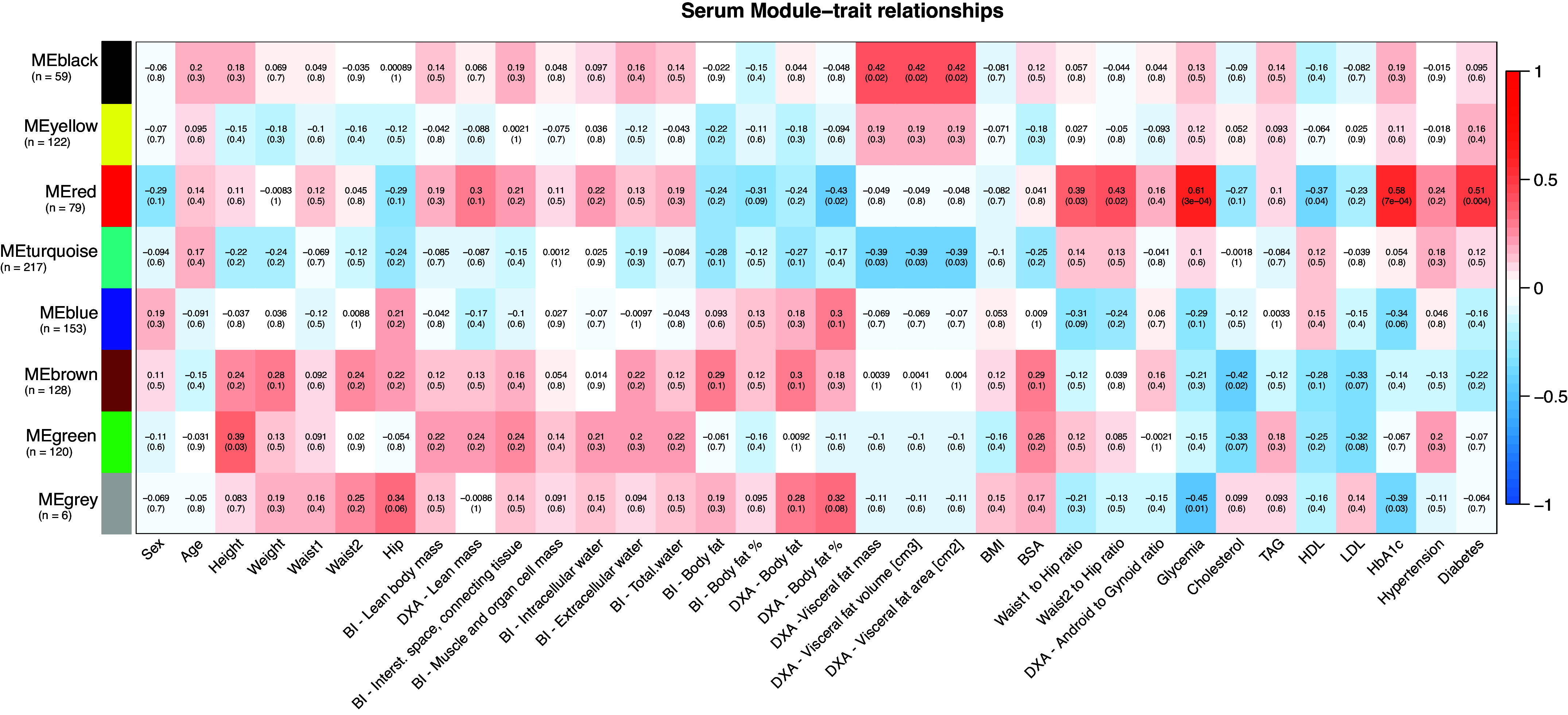
Serum module-traits relationships. Relationships between serum module eigengenes derived from weighted gene coexpression network analysis (WGCNA) and selected clinical variables, along with corresponding Pearson correlation *P* values. Strong positive correlations are represented in red, while strong negative correlations are in blue; numbers within individual cells indicate the correlation coefficient and (*P* value). Significant module-trait correlations (*P* value < 0.05) with an absolute correlation value >0.5 are highlighted in bold. Module sizes are provided in brackets. Waist1 refers to waist circumference measured at the inferior margin of the ribs, and Waist2 denotes measurements at the umbilical level. BI, bioimpedance; BMI, body mass index; BSA, body surface area; DXA, dual-energy X-ray absorptiometry; HDL, high-density lipoprotein; LDL, low-density lipoprotein; TAG, triacylglycerols.

The outcomes of the weighted gene coexpression network analysis (WGCNA) unveiled a division of the SAT, VAT, and serum proteomes into 9, 11, and 7 modules, respectively. These modules encapsulate proteins with plausible coexpression or functional associations. The functional enrichment analysis conducted on these modules revealed the presence of highly conserved biological processes and protein complex structures (details available in Supplemental File S13). To delve into the potential associations between proteome structure and clinical attributes, we carried out a correlation analysis between the modules and the clinical traits (Figs. 5, 6, and 7).

To expand upon the link between clinical traits and the proteomes, we scrutinized individual proteins within modules displaying the most robust correlations with clinical variables. Given the considerable intercorrelation among certain clinical variables (Supplemental File S14), we logically grouped these traits. Subsequently, the correlation analysis unveiled a multitude of significant protein-trait associations (Supplemental File S15, *A*–*C*). To enhance the focus on the most insightful correlations, we performed an enrichment analysis of the significantly associated proteins, focusing on those with the highest correlation coefficients and those that hold functional relevance to the underlying pathophysiology of obesity.

Importantly, it should be noted that the proteins listed in *Weighted Gene Coexpression Network Analysis* are not exhaustive; rather, they represent selected examples showcasing the most notable correlations with traits and/or their potential contribution to obesity’s pathophysiology.

#### WGCNA—sex-dependent protein expression patterns in adipose tissue.

Sex plays a significant role in energy metabolism and fat storage. WGCNA results highlighted distinct protein expression patterns: energy metabolism-related proteins (SAT Blue and Brown modules) exhibited higher expression in females. In contrast, translation-related proteins (SAT Red and Yellow modules) were more pronounced in males. In addition, males showed increased expression of proteins involved in vesicle-mediated transport (VAT Black module). Specifically, XRN2 and STARD5 (SAT), as well as FOSL2 and SIGMAR1 (VAT), were more highly expressed in males. Among females, higher expression was noted for GYG2 in both AT depots, PPAP2A (SAT), and AGT and CA3 (VAT).

The LIMMA analysis reinforced these findings (Supplemental File S5). It indicated upregulation of DHRS3, EMC2, EIF3J, GYG2, CKB, PALMD, QDPR, and PPAP2A in female SAT samples and PNPLA7 in male SAT samples, all with Log2FC > 1. Female VAT samples demonstrated upregulation of CA3 and AGT (Log2FC > 1), whereas no significant protein upregulation was found in male VAT samples.

#### WGCNA—SAT and VAT relation to lean mass and related variables.

Strong intercorrelations were evident between BI and DXA lean mass, along with BI-derived values related to interstitial space and connective tissue, muscle and organ cell mass, intracellular water, extracellular water, and total water. The SAT Turquoise and Yellow modules, as well as the VAT Black module, exhibited significant positive correlations with these traits. Notably, both tissues demonstrated module enrichment in proteins associated with extracellular exosomes and vesicle-mediated transport. The SAT modules additionally revealed enrichment in metabolic proteins, particularly those involved in protein and RNA metabolism.

Conversely, a negative correlation with these traits was observed for the SAT Blue module and VAT Brown and Pink modules. The SAT Blue and VAT Brown modules exhibited enrichment in proteins involved in the generation of precursor metabolites and energy through the mitochondrion and related processes. The VAT Pink module displayed enrichment in proteins regulating extracellular vesicles and the complement cascade. However, the negative correlation of VAT modules with these traits was milder compared with that observed for SAT.

Proteins in SAT that positively correlated with lean mass and intercorrelated traits demonstrated enrichment for ECM organization, integrin-cell surface interactions, and metabolic processes, including glucose metabolism. Similarly, VAT proteins showing a positive correlation with lean mass and related traits were enriched in the innate immune system, neutrophil degranulation, and vesicle-mediated transport. Both SAT and VAT exhibited a negative correlation with lean mass for proteins associated with metabolism, particularly the citric acid cycle and respiratory electron transport, with SAT proteins demonstrating a stronger association. Furthermore, SAT showed a negative correlation with lean mass for proteins involved in fatty acid metabolism and lipid metabolism.

#### WGCNA—SAT and VAT relation to weight, waist circumferences, height, BMI, and BSA.

Weight, waist circumferences, BSA, and height exhibited comparable correlations to lean mass and related traits, although these associations were generally weaker and displayed certain distinctions. Unlike these traits, BMI did not show any significant association with the SAT or VAT modules, resulting in limited enrichment of proteins with significant correlations.

In SAT, proteins with positive correlations to these traits were enriched in processes related to hemostasis, platelet activation, signaling, and regulation of the complement cascade. Conversely, VAT proteins displayed a consistent positive correlation trend with proteins linked to the innate immune system, neutrophil degranulation, and antigen processing-cross presentation. Remarkably, this enrichment was particularly significant in relation to waist circumferences.

The negative correlations between SAT proteins and these traits indicated a reduced expression of proteins engaged in protein metabolism, including translation and metabolism of amino acids and derivatives. In addition, proteins associated with mitochondrial energy metabolism, including the citric acid cycle and respiratory electron transport, cristae formation, and fatty acid β-oxidation of unsaturated fatty acids, exhibited decreased expression. A comparable reduction in the expression of the citric acid cycle and respiratory electron transport proteins was also observed in VAT proteins, particularly concerning waist circumferences.

#### WGCNA—SAT and VAT relation to waist-to-hip and android-to-gynoid ratios.

The ratios used to assess body fat distribution, such as the W1TH, W2TH, and the DXA android-to-gynoid (A/G) ratio, reveal consistent expression patterns. Although the modules associated with these ratios displayed varying degrees of significance concerning the traits, the enrichment analysis of correlated proteins uncovered similar processes. These parameters underscore the disparities between SAT and VAT, where correlations to the traits are comparatively weaker in VAT’s protein expression patterns.

In SAT, proteins related to the energy metabolism, encompassing the citric acid cycle and respiratory electron transport, and fatty acid metabolism exhibit reduced expression with escalating abdominal obesity. In contrast, the expression of SAT proteins involved in the innate immune system, like those linked to neutrophil degranulation and unspecific metabolism, increases with abdominal obesity. Notably, proteins engaged in ECM organization, including the small leucine-rich proteins LUM and BGN, also displayed a positive correlation.

Similar pathways and reactions displaying positive correlations with these ratios were observed in VAT proteins. However, the negative correlation observed in VAT was comparatively milder than in SAT. The regulation of insulin-like growth factor transport and uptake by insulin-like growth factor binding proteins emerged as the most significantly enriched pathway among proteins exhibiting negative correlation. This observation was particularly pronounced in connection with A/G and W2TH ratios. Although the proteins with the strongest correlations were analogous for both waist-to-hip (WTH) ratios, the A/G ratio exhibited distinct correlations.

#### WGCNA—SAT and VAT relation to body fat content.

Another cluster of clinical traits demonstrated a connection with body fat mass and its proportion, assessed through both BI and DXA, as well as hip circumference. Specifically, body fat mass negatively correlated with lean mass and related traits, particularly when quantified via BI. The body fat percentage exhibited stronger correlations with the SAT and VAT protein modules, and the enrichment of the correlating proteins displayed intricate biological pathways and processes. However, body fat measurements from DXA yielded weak enrichments with no significant association with any protein module. Total body fat, measured by BI, revealed a negative correlation between immune system-related proteins and protein metabolism in SAT.

Meanwhile, the body fat proportion displayed negative correlations in both SAT and VAT with proteins linked to the immune system, innate immunity, neutrophil degranulation, and disease. In SAT, positive correlations emerged in metabolic processes, such as the citric acid cycle and respiratory electron transport, as well as lipid metabolism. In contrast, VAT demonstrated positive correlations with proteins involved in platelet activation, signaling, and aggregation, along with protein metabolism. Notably, the proteins displaying the strongest correlations varied among these traits.

#### WGCNA—SAT and VAT relation to visceral fat, age, glycemia, and HbA1c.

Visceral fat mass, area, and volume exhibit strong intercorrelations, forming a unified entity. An upward trend in visceral fat mass was apparent with advancing age and elevated glycemia and HbA1c levels. This trend resonated in the associated proteomic correlations within both SAT and VAT. Specifically, proteins linked to ECM organization, ECM proteoglycans, and axon guidance displayed increasing expression in SAT with increasing visceral fat mass. Similarly, age, glycemia, and HbA1c levels exhibited this trend, whereas enrichment analysis unveiled additional involvement of proteins in the innate immune system, vesicle-mediated transport, and cellular response to stress. Similar enrichments were observed in VAT, encompassing proteins related to the immune system and vesicle-mediated transport. Conversely, a negative correlation to increasing visceral fat mass was noted for proteins involved in metabolism, particularly mitochondrial energy metabolism processes.

The decrease in the expression of functionally related proteins was more pronounced in VAT, where 47 proteins (false discovery rate, FDR = 1.11*e*-41) enriched pathways related to the citric acid cycle and respiratory electron transport, contrasting with SAT’s 11 proteins (FDR = 0.0037). While akin patterns of enriched pathways and processes emerged, the proteins exhibiting the strongest correlations varied across these traits.

#### WGCNA—SAT and VAT relation to cholesterol, HDL, LDL, and TAG.

Cholesterol and LDL blood levels demonstrated strong intercorrelations, and a similar trend was observed for HDL. In SAT, a positive correlation linked cholesterol and LDL levels with proteins engaged in chylomicron assembly (APOA1, APOA2, APOB, APOC1, and APOM). Conversely, negative correlations emerged between SAT and cholesterol/LDL levels and proteins involved in translation, positive epigenetic regulation of ribosomal RNA expression, and transport.

In VAT, positive correlations and enrichment of associated proteins were noted in cap-dependent translation initiation, formation of a pool of free 40S subunits, and translation termination. Conversely, negative correlations in VAT between cholesterol/LDL levels and proteins were associated with signaling by Rho GTPases, axon guidance, and the immune system. However, the overall enrichment of these traits in VAT was modest.

Furthermore, significant associations were observed between SAT modules and HDL. Correlated proteins exhibited a weak association with programmed cell death, metabolism (positive correlation), as well as disease and axon guidance (negative correlation). Similarly, HDL levels correlated with VAT proteins involved in regulating insulin-like growth factor transport and uptake by insulin-like growth factor binding proteins, protein metabolism, and plasma lipoprotein assembly (APOA1, APOA4, APOC1, APOC3, APOE, APOL1). In contrast, a negative correlation was found between HDL levels and proteins associated with the innate immune system, neutrophil degranulation, and proteasome substrate cleavage. TAG levels did not correlate with any modules; however, a positive correlation emerged between TAG and VAT proteins in LDL clearance.

#### WGCNA—SAT and VAT relation to hypertension and T2D.

Patients diagnosed with hypertension exhibited elevated expression of immune system-associated proteins in both SAT and VAT. Alongside this, a notable reduction in protein expression was evident in metabolic processes, particularly the citric acid cycle and respiratory electron transport, mitochondrial fatty acid β-oxidation, and other energy metabolism-related processes in both tissues. This association was more pronounced in VAT. In SAT, increased expression of proteins like SIGLEC1, CMAS, MRRF, PDIA5, and CD163 was observed, whereas in VAT, proteins such as ATL1, TNFAIP2, F13A1, UTS2, TBXAS1, ENTPD1, or VASP displayed heightened expression. Conversely, decreased expression was linked to proteins such as WASF2, TEP1, CCDC18, FMOD, and SLC25A10 in SAT, and to proteins such as ACOX2, ACAD9, ALDH6A1, PRDX3, UQCRC1, ATP5B, and ATP5A1 in VAT.

For patients with T2D, reduced expression of metabolic proteins was observed in both SAT and VAT. Notably, a more pronounced enrichment of the citric acid cycle and respiratory electron transport was observed in VAT protein expression. In addition, elevated expression of proteins associated with infectious diseases and cellular responses to stress was observed in both ATs. In SAT, increased expression of ECM proteoglycans (LUM, OGN, BGN, ASPN) was also associated with T2D. In the SAT of patients with T2D, increased expression of proteins like PPP1R14B, MATN2, COL6A3, PEMT, COL6A1, BGN, SERPINE1, VPS33B, COL6A2, or COX8A was noted. Conversely, increased expression of proteins such as PI16, AIF1L, COMMD3, RPN1, HOOK2, ITGAM, ANXA5, and ANGPTL2 was observed in VAT of patients with T2D. In contrast, decreased expression in patients with T2D was observed for proteins like CREBBP, NFCI, CTBP2, ALDOC, S100A1, FASN, and STAT5B in SAT and for proteins such as UQCC2, CYCS, HADH, ARG1, SELENBP1, and PRD3 in VAT.

### Obesity Signatures in Serum

The WGCNA identified seven distinct modules based on the proteome expression patterns (Fig. 7). The Red module demonstrated the most significant associations with clinical traits. It was enriched with proteins involved in the binding and uptake of ligands by scavenger receptors, the insulin-like growth factor ternary complex, and the lectin pathway of complement activation. Notably, the Red module showed a positive correlation with WTH ratios, glycemia, HbA1c levels, and T2D, whereas a negative correlation was evident with DXA body fat percentage and HDL levels. Within this module, prominent positive correlations with the WTH ratios were seen for CDHR2, NCAM1, ALDHA1, CD163, CD81, CDH2, IL1R2, FABP1, and ALDOB, whereas negative correlations included PRDX1, AZU1, PLXND1, IGFALS, MPO, SERPINA6, and SAA2. The A/G ratio did not show any significant correlation to the serum modules, but a positive correlation was observed with CPA4, NCAM1, CDH2, IL1R2, and a negative correlation with SIRPA, LAMC1, PKM, and SAA2.

The Red serum module demonstrated the most significant positive correlation with glycemia and HbA1c levels, particularly for FABP1, ADAMTSL2, ALDOB, HSPA5, SOD2, CD163, LUM, and SERPINF1. Conversely, XYLT2, CCN2, SPTA1, APOF, MMP8, and ANGPTL3 showed negative correlations. This module also exhibited a positive correlation with T2D, with notable associations for FABP1, ADAMTSL2, SOD2, QSOX, ALDOB, L1CAM, PGM1, CSF1R, ALDH1A1, CD163, and LUM. Lower expression in patients with diabetes was evident for SCPEP1, S100A11, LEP, PEBP1, SHGB, and APOF. No significant correlation was observed between the serum module and hypertension. Still, an increased expression of SVEP1, NRP1, RNASE1, QSOX1, and GKN1, along with decreased expression of XYLT2, CFH, LEP, and CETP serum levels were found in patients with hypertension.

Furthermore, the serum Red module negatively correlated with HDL levels, with proteins like VCP, FCN3, TALDO1, HSP90AA1, and PGAM1 showing significant associations. The most significant positive correlation with HDL levels was observed for proteins NOTCH3, CD248, GSN, SHBG, and APOF. In addition, the serum Brown module negatively correlated with cholesterol levels, enriched with proteins associated with humoral immune response, complement activation, and neutrophil degranulation. The proteins with the most significant negative correlation to cholesterol levels were GDI2, ENO1, AMY2A, PGD, NTRK2, COL3A1, and SERPINA1, which also showed a negative correlation to LDL levels. On the other hand, positive correlations with cholesterol and LDL levels were observed for LRP1, APOF, APOM, APOC2, APOB, and APOC1. In addition, positive correlations with TAG levels were found for proteins VCP, CAMP, IL1RAP, CD81, and HSP90AA1, whereas negative correlations were observed with PSMA4, AMY2A, HGFAC, NTRK2, and ITGAM serum levels.

The serum Red module also demonstrated a negative correlation with DXA body fat percentage, with the most significant correlations found with CDON, GPT, CPA1, CHGA, and ALDH1A1. Conversely, positive correlations were observed with proteins CALM3, LEP, SAA2, and multiple immunoglobulins. Although no significant associations were found between the BI and DXA total body fat and BI body fat percentage with any serum module, the proteins with the most significant correlations were similar. For BI total body fat, significant negative correlations included CPA1, HSPD1, LUM, and APOA4, whereas significant positive correlations included CALML3, LEP, CRP, and GPD1. For BI body fat percentage, significant negative correlations were observed with HSPD1, ANG, ROBO4, and STAB1, and significant positive correlations included NBL1, XYLT2, CALM3, and LEP. For DXA total body fat, significant negative correlations were evident with CDON, CPA1, GPT, CHGA, NOTCH1, APOA4, and NEGR1, whereas significant positive correlations included CALML3, S100A14, LUM, HSPB1, CRP, GPD1, and LEP.

Two serum modules showed significant associations with visceral fat variables. The serum Black module, enriched in proteins associated with the collagen-containing ECM, plasma lipoprotein particles, and acute phase response, exhibited a positive correlation. The most significant positive correlations with visceral fat mass were seen for HTRA1, CALU, haptoglobin, APMAP, SAA4, and multiple complement proteins. Conversely, the serum Turquoise module, enriched in proteins related to extracellular space, cell migration, ECM organization, ECM proteoglycans, and regulation of IGF transport and uptake by IGFBP, showed a negative correlation. The most significant negative correlations were observed for SNCA, IGFALS, CAP1, PRDM8, and FETUB serum levels.

No other traits, except height, were significantly associated with the serum modules. Height positively correlated with the serum Green module enriched in proteins involved in gluconeogenesis. Notably, VCP showed the most significant positive correlation with all the lean mass and related traits, whereas NBL1 exhibited a negative correlation. Positive correlations with BMI were noted for CEP192, CALML3, NCL, CRP, CD48, GPD1, and SAA2, whereas negative correlations were observed for APOA4 and CPA1 levels. In addition, positive correlations emerged between the BSA and CALML3, VCP, PRDX6, GPD1, and HSPB1 serum levels, with negative correlations observed for NOTCH1, L1CAM, and IGFBP1. Although no significant correlation emerged between serum modules and sex, a heightened expression pattern of heat shock proteins (HSP90, HSP90AA1, HSPA4, HSPA8, and HSPD1) was noted in male serum samples. The sole differentially expressed protein between sexes identified through LIMMA differential expression analysis was PSMA7, which showed upregulation in male samples (Supplemental File S6). Finally, positive correlations with age were identified for RNASE1, QSOX1, CTSL, PRDM8, LUM, CST3, ANG, and POSTN, whereas negative correlations were found between SNCA, IGFALS, CAP1, PRDM8, and FETUB serum levels.

## DISCUSSION

Obesity has become a global health concern. Understanding its underlying molecular mechanism is crucial for improving diagnosis and treatment. Proteomic profiling has emerged as a promising approach to unravel the complex network of molecular events underlying obesity. This article presents the most comprehensive proteomic description of SAT, VAT, and serum of severely obese patients to date (33, 34). We aim to shed new light on the molecular basis of obesity while also identifying potential biomarkers for clinical use.

By employing the BoxCar MS data acquisition method (20) in conjunction with the spectral library of adipocyte proteomes, along with DIA MS of serum samples after the depletion of the top 14 most abundant proteins, we successfully identified 7,284 proteins in AT samples and 1,206 in serum samples. This innovative approach allowed us to effectively overcome the challenges posed by the wide dynamic range imposed by blood-borne proteins and attain a significantly deeper coverage of the AT and serum proteome compared with the previous studies (35–38). Moreover, we collected high-quality anthropometric data using both BI and DXA techniques. This enabled us to explore differences in protein expression based on body composition and identify distinct protein expression patterns associated with specific traits.

Our differential expression analysis revealed noteworthy differences between VAT and SAT. The outcomes highlight the significantly greater complexity of SAT’s molecular profile than VAT’s. This distinction is evident through a higher count of differentially expressed proteins and a broader array of pathways identified in SAT. Specifically, our study reveals elevated expression of proteins associated with ECM remodeling, cellular stress response, and inflammation in SAT compared with VAT. Moreover, SAT exhibited increased expression of proteins involved in vesicular transport, exosome biogenesis, and lipid metabolism. Conversely, our analysis identified proteins with elevated expression in VAT primarily engaged in mitochondrial energy metabolism and RNA metabolism, including translation.

These observations align with our previous study of the adipocyte proteome (8). When comparing the upregulated proteins across adipocytes and tissue proteomes, robust preservation of expression patterns emerged in VAT for proteins linked to mitochondrial energy metabolism and translation. In contrast, overlapping upregulated proteins in SAT highlighted processes related to lipid metabolism, extracellular exosomes, and ECM organization. This substantial concurrence in differentially expressed proteins between adipocytes and ATs, especially in VAT, further underscores the integral role of adipocytes in shaping AT’s intricate molecular landscape.

Furthermore, our analysis revealed proteome signatures demonstrating expression correlations with obesity-related clinical traits. This underscores the substantial pathophysiological changes that unfold within AT during the progression of severe obesity. Despite this study encompassing a relatively homogeneous group of patients with severe obesity eligible for bariatric surgery, our anthropometric measurements highlighted significant diversity in subphenotypes, notably reflected in the proteome signatures. Importantly, our findings highlighted that BMI inadequately translates into discernible proteome patterns reflecting the tissue’s underlying physiological state, particularly in cases of severe obesity.

Collectively, our findings indicate that in severe obesity, the lipid turnover capacity of SAT becomes overwhelmed, potentially resulting in ectopic fat accumulation, chronic inflammation, and fibrosis—fundamental drivers of obesity-related metabolic disorders. Within AT, collagens emerge as a predominant ECM constituent, and their accumulation can trigger fibrotic changes, increase tissue rigidity, reduce the expandability of AT, and ultimately contribute to IR (39). Our study demonstrates that nearly all identified collagen variants exhibit heightened expression in SAT. Among these collagens, collagen VI (COL6A1, A2, A3) stood out, showing positive correlations with glycemia, HbA1c levels, visceral fat mass, and negative correlations with total fat mass. Moreover, its expression in SAT showed elevated levels in patients with T2D. Previous studies have shown the direct impact of collagen VI on adipocytes’ ability to expand (40), and its expression displays a positive association with visceral fat mass (41). Beyond this, collagen VI has been implicated in promoting fibrotic changes, amplifying tissue inflammation, and exacerbating IR (42–44).

Furthermore, the linkage of collagen VI to fibrotic processes and IR has also been reported in relation to CD163+ cell accumulation (45). In congruence with prior research, our findings reinforce this connection. We found a significant correlation between CD163 levels in SAT and key parameters, including visceral fat mass, glycemia, HbA1c levels, T2D, and hypertension. In addition, serum levels of CD163 displayed an increase with T2D. In addition, periostin, a protein involved in tissue remodeling and fibrosis, emerged among the most significantly upregulated proteins within SAT. Studies have shown that its depletion attenuates AT fibrosis, enhances insulin sensitivity, reduces macrophage infiltration and crown-like structure formation, and mitigates ectopic lipid accumulation (46, 47).

Another set of ECM components exhibiting distinct expression patterns between the tissues consists of small leucine-rich proteoglycans (SLRPs). In a previous study, we highlighted elevated expression levels of mimecan (OGN), lumican (LUM), asporin (ASPN), and biglycan (BGN) in visceral adipocytes (8). The growing evidence underscores the pivotal role these molecules play in IR and metabolic inflammation (48–50). Although SLRPs are significantly more abundant in VAT, our novel findings propose an intriguing connection: within SAT, SLRP expression increases with age, visceral fat mass, glycemia, and HbA1c levels—aligning with the development of T2D. Specifically, we observed a significant association between the biglycan expression, renowned for scaffolding collagen fibrils and facilitating cell signaling, and T2D in SAT. Biglycan has also demonstrated the capacity to activate the expression of inflammatory genes in adipocytes, thereby contributing to IR (51). Similarly, lumican—recognized for its role in the repairing process of collagen-rich connective tissue (52), has been shown to play a role in AT inflammation and IR (53). Abnormal accumulation of lumican in SAT modifies collagen I organization, impairs adipogenesis, and induces stress responses in adipocytes, ultimately contributing to the IR (36). Of note, our study also unveils a correlation between serum lumican levels and age, along with elevated levels in the serum of patients with T2D, indicating its potential as a target for biomarker discovery. The notable shifts within SAT could signify a transient metabolic phenotype that may underlie the eventual development of T2D over time.

Cell-cell and cell-ECM interactions are crucial in orchestrating AT inflammation and IR. Among the proteins involved in these interactions, CD44, which is upregulated in SAT, has been linked to AT inflammation and IR (54). However, our study did not observe any association between CD44 and T2D, but we found a positive correlation of glycemia and HbA1c levels with CD44 expression within SAT. Another noteworthy protein involved in cell-ECM interactions, CD248 (endosialin), also displayed elevated expression in SAT. This protein is recognized for its role in the positive regulation of proinflammatory/profibrotic pathways and mediating part of the AT response to hypoxia (55). CD36, also referred to as fatty acid translocase, facilitates the transport of free fatty acids into the AT and binds to many ligands, including lipoproteins and collagen. Its higher expression in SAT can contribute to macrophage infiltration and IR (56). Elevated numbers of antigen-presenting cells in SAT align with higher expression of major histocompatibility complex II (MHC II), a core component for antigen presentation and activation and maturation of CD4+ T cells. Such cellular dynamics can further fuel AT inflammation and IR (57–59).

Moreover, integrins, a family of transmembrane receptors essential for mediating cell-matrix interactions and signaling pathways in adipocyte differentiation, tissue inflammation, and insulin sensitivity, exhibited heightened expression, primarily within SAT. Our observations revealed an increased expression of upregulated SAT integrins, including α2 b, α3, and αV, associated with increasing visceral fat mass. Furthermore, integrins such as β2 (CD18) are crucial for macrophage migration, influencing the equilibrium between pro- and anti-inflammatory macrophages in AT. High expression of CD18 on macrophages prevents their amoeboid migration, thereby retaining them at inflammatory sites and promoting chronic inflammation (60, 61). Although CD18 expression was significantly higher in SAT, its expression in VAT was decreasing with increasing visceral fat mass. This shift might signify an imbalance of pro- and anti-inflammatory macrophages in AT. One of four CD18 coupling proteins, integrin αM (CD11b), was associated with increased expression in VAT of patients with T2D. Previous studies have shown that reduced CD11b expression attenuates IR and improves glucose tolerance by limiting proliferation and alternative activation of AT macrophages (62).

Drawing upon recent evidence, fibrosis and inflammation within AT are closely interwoven with alterations in mitochondrial metabolism—a critical regulator of cellular energy homeostasis (63). Impaired mitochondrial oxidative metabolism is a hallmark of obese AT, observed across animal and human studies, irrespective of glucose tolerance status (64–67). Research has reported downregulated mtDNA, decreased abundance of mitochondrial proteins, and impaired oxidative phosphorylation capacity in SAT (64, 68, 69). Our observations echo these trends, revealing lower expression of mitochondrial complex I, III, IV, and related proteins in SAT compared with VAT. Furthermore, our findings align with prior studies showcasing impaired fatty acid oxidation within SAT (70). Reduced expression of mitochondrial metabolism-related proteins in VAT has also been linked to aging and T2D (65). Our data reinforce these trends, illustrating decreases in these proteins as visceral fat mass or glycemia rises, along with concurrent increases in protein expression related to the immune system. This downregulation of mitochondrial-related proteins in VAT was also associated with hypertension and T2D.

These observations underscore the pivotal role of mitochondria within AT and imply a potential role of VAT as a safeguard against insufficient oxidative capacity in SAT. Yet, this exposes VAT to escalated oxidative activity, potentially resulting in an augmented generation of reactive oxygen species and oxidative stress that ultimately fuels inflammation. Hence, rooted in our observations, it becomes evident that maintaining a delicate balance between fatty acid oxidation and free fatty acid flux to other tissues is paramount—without disrupting the redox equilibrium and triggering AT inflammation.

Arner et al. (71) have previously outlined the significance of high storage coupled with limited TAG removal in the context of fat tissue accumulation and obesity. As research unfolds, adipocyte lipid turnover emerges as a fresh avenue for targeting the prevention and management of metabolic diseases. It has been shown that in SAT, lipid turnover is already attenuated during the overweight state, whereas in VAT, this reduction is only evident in cases of severe obesity (72). In this context, our findings plausibly substantiate the compensatory role of VAT in response to the overloaded SAT.

Our results expose an elevated expression of endoplasmic reticulum (ER) stress-related proteins in SAT, likely stemming from excessive lipid accumulation, adipocyte expansion, and fibrosis. Specifically, in SAT, we identified heightened expression of IRE1α—a sensor for unfolded proteins. This might lead to translational inhibition and the induction of ER-associated degradation (ERAD) via proteasome activation (73), a process similarly heightened within SAT. Conversely, VAT exhibited increased expression of proteins in protein synthesis, indicative of tissue expansion and its accompanying metabolic requisites. This elevation prompts an augmented mitochondrial metabolism to meet the heightened energy demands aligned with accelerated protein synthesis. Furthermore, the upsurge of heat shock proteins and other proteostasis-maintaining proteins within VAT signifies an adaptive response to the amplified metabolic pressures and cellular stress stemming from its possible compensatory role in lipid storage and energy homeostasis (74).

Notably, we observed nearly exclusive expression of intelectin-1 (omentin-1) in VAT—an adipokine recognized for its anti-inflammatory attributes. Omentin-1, known to promote proliferation, inhibits apoptosis, and increases the secretion of angiogenic cytokines (75), has also been shown to reduce ER stress, oxidative stress, and NO production (76). Consequently, omentin-1 could play a pivotal role in sustaining the metabolically healthy expansion of VAT, thereby supporting its possible compensatory role. Yet, when the capacity of SAT is overwhelmed, and VAT’s adaptive mechanisms are insufficient, it may ultimately lead to ectopic lipid deposition, IR, and the adverse outcomes associated with obesity. Deciphering the intricate interplay between SAT and VAT in maintaining energy homeostasis and proteostasis could potentially unveil novel therapeutic strategies for addressing obesity and related metabolic disorders.

In addition, we aimed to identify serum protein markers capable of reflecting tissue expression or metabolic states in patients. Intriguingly, we discovered that serum adiponectin and leptin levels were significantly correlated with SAT and VAT expression, respectively. Although both proteins are known for their pivotal roles in the development of obesity (77, 78), their potential to predict specific outcomes or serve as reliable biomarkers remains limited (78). Adiponectin acknowledged for its anti-inflammatory and insulin-sensitizing effects, has previously been linked to higher expression in SAT—consistent with our observation (79, 80). Moreover, we also observed decreasing adiponectin expression within both SAT and serum with increasing visceral fat mass. In contrast, leptin’s expression in VAT and serum does not mirror such correlations with clinical traits.

Furthermore, SEMA3C emerged as one of the most profoundly correlated proteins with SAT. Although it was not associated with any clinical traits in our study, previous research has identified SEMA3C as an adipokine correlating positively with AT hypertrophy and fibrosis (81). Intriguingly, its serum expression decreases with exercise-induced weight loss (82), rendering SEMA3C a plausible candidate for indicating SAT capacity. Moreover, our findings highlight a substantial serum linkage with elevated fatty acid-binding protein 1 (FABP1) expression in patients with T2D, regardless of tissue expression. FABP1’s correlation extends further, aligning with glycemia, HbA1c levels, and abdominal obesity delineated through WTH ratios. Previous studies have associated FABP1 with nonalcoholic fatty liver disease and nephropathy in patients with T2D (83, 84), effectively underscoring its potential as a biomarker for metabolic dysfunction.

Our study does come with some limitations that we want to acknowledge. The sample size, involving 32 patients, is diverse in terms of clinical parameters, but it may not provide sufficient statistical strength to identify subtle associations, potentially leading to false positive findings. In addition, our patient cohort consists mainly of white individuals of Central European origin, limiting the broader applicability of our conclusions to other populations. The study also focuses only on a specific group of patients with severe obesity eligible for bariatric surgery. It is important to acknowledge that the practice of short-term fasting before surgery could have potentially influenced the expression of certain proteins in the proteomes, possibly reflecting the fasting state. This highlights the need for future studies across diverse ethnic and phenotype groups. Another aspect to consider is the impact of the serum preparation and the depletion process, which can introduce alterations to the natural composition of peripheral blood proteomes. Consequently, our findings might not fully mirror the inherent state of the peripheral blood proteome in the context of severe obesity and its associated comorbidities. Finally, due to space constraints, we were unable to present all the data within this article. However, we have made all our data accessible, encouraging collaborative efforts and facilitating more in-depth analyses. This approach fosters the generation of additional insights within the realm of obesity-related proteomics research, ultimately magnifying the significance of our work.

Despite these limitations, our study has several notable strengths, delivering insightful perspectives into the intricate interplay between SAT and VAT within the context of severe obesity. One of the most prominent strengths is our utilization of state-of-the-art technology and progressive proteomics MS analysis techniques. By integrating advanced imaging modalities like DXA to comprehensively delineate body composition and the untargeted analysis of paired AT and serum samples of patients with severe obesity, we were able to conduct the most comprehensive proteomics profiling to date. In addition, our study features an equal distribution of sexes, ensuring that our findings apply to both males and females. From a clinical standpoint, the significance of our study is underscored by the inclusion of patients with diagnosed T2D and hypertension, allowing for the exploration of the intricate interconnection between proteomic profiles and these prevalent comorbidities. Furthermore, using WGCNA to identify modules of correlated proteins and clinical variables increases the robustness of our findings and reduces the likelihood of false positives. As such, our study offers further evidence highlighting the importance of research into the metabolic properties of AT in various anatomical locations across the body.

### Conclusions

Our study provides the most comprehensive proteomic description of SAT, VAT, and serum of patients with severe obesity to date. The proteomic analysis revealed signs of fibrosis and inflammation in the SAT, suggesting SAT overload and lipid overflow. This seems to be reflected by an adaptive VAT enlargement, which shows significant upregulation of proteins related to energy metabolism and protein synthesis. The overflow of SAT could contribute not only to VAT expansion but also potentially result in ectopic fat storage, IR, and the development of T2D. This study underlines the importance of understanding the intricate balance between SAT and VAT in maintaining energy homeostasis and proteostasis. Gaining a deeper understanding of these mechanisms emphasizing SAT capacity may pave the way for developing novel therapeutic strategies targeting obesity and its associated metabolic disorders. Further research is warranted to validate and expand upon these findings in diverse populations and to explore the implications of these molecular mechanisms in obesity and related metabolic complications.

## DATA AVAILABILITY

The MS proteomics data and the search results have been deposited to the ProteomeXchange Consortium via the PRIDE (24) partner repository with the data set identifier PXD041721 (AT samples) and PXD041750 (serum samples). The R scripts are available upon request.

## SUPPLEMENTAL DATA

10.6084/m9.figshare.22825532 Supplemental Files S1–S15: https://doi.org/10.6084/m9.figshare.22825532.

## GRANTS

This study was supported by a Specific University Research Grant MUNI/A/1343/2022 provided by the Ministry of Education, Youth and Sports of the Czech Republic, CETOCOEN PLUS Project CZ.02.1.01/0.0/0.0/15_003/0000469 (CEP: EF15_003/0000469), RECETOX research infrastructure (Ministry of Education, Youth and Sports of the Czech Republic) under Grant No. LM2023069, CETOCOEN EXCELLENCE Teaming 2 project supported by Horizon2020 under Grant No. 857560 and the Ministry of Education, Youth and Sports of the Czech Republic under Grant No. CZ.02.1.01/0.0/0.0/17_043/0009632. CIISB, Instruct-CZ Centre of Instruct-ERIC EU consortium, funded by MEYS CR infrastructure Project LM2023042 and European Regional Development Fund-Project “UP CIISB” under Grant No. CZ.02.1.01/0.0/0.0/18_046/0015974, is gratefully acknowledged for the financial support of the measurements at the CEITEC Proteomics Core Facility. Computational resources were provided by the e-INFRA CZ project under Grant ID:90140, supported by the Ministry of Education, Youth and Sports of the Czech Republic. This work was supported by the European Union’s Horizon 2020 research and innovation program under Grant Agreement No. 857560.

## DISCLAIMERS

This publication reflects only the author’s view, and the European Commission is not responsible for any use that may be made of the information it contains.

## DISCLOSURES

No conflicts of interest, financial or otherwise, are declared by the authors.

## AUTHOR CONTRIBUTIONS

P.H., J.K., M.B., M.P., P.H., D.P., Z.Z., and J.B.-V. conceived and designed research; P.H., M.B., M.P., P.H., and D.P. performed experiments; P.H., D.K., and D.P. analyzed data; P.H., J.K., and J.B.-V. interpreted results of experiments; P.H. and D.K. prepared figures; P.H. drafted manuscript; J.K., D.P., Z.Z., and J.B. edited and revised manuscript; P.H., J.K., D.K., M.B., M.P., P.H., D.P., Z.Z., and J.B.-V. approved final version of manuscript.

## References

[1] The GBD 2015 Obesity Collaborators. Health effects of overweight and obesity in 195 countries over 25 years. N Engl J Med 377: 13–27, 2017. doi:10.1056/NEJMoa1614362. 28604169 PMC5477817

[2] Fox CS, Massaro JM, Hoffmann U, Pou KM, Maurovich-Horvat P, Liu C-Y, Vasan RS, Murabito JM, Meigs JB, Cupples LA, D'Agostino RB, O'Donnell CJ. Abdominal visceral and subcutaneous adipose tissue compartments. Circulation 116: 39–48, 2007. doi:10.1161/CIRCULATIONAHA.106.675355. 17576866

[3] Karlsson T, Rask-Andersen M, Pan G, Höglund J, Wadelius C, Ek WE, Johansson Å. Contribution of genetics to visceral adiposity and its relation to cardiovascular and metabolic disease. Nat Med 25: 1390–1395, 2019. doi:10.1038/s41591-019-0563-7. 31501611

[4] Reijrink M, de Boer SA, Spoor DS, Lefrandt JD, Lambers Heerspink HJ, Boellaard R, Greuter MJ, Borra RJH, Hillebrands J-L, Slart RHJA, Mulder DJ. Visceral adipose tissue volume is associated with premature atherosclerosis in early type 2 diabetes mellitus independent of traditional risk factors. Atherosclerosis 290: 87–93, 2019. doi:10.1016/j.atherosclerosis.2019.09.016. 31604171

[5] Tanaka T, Kishi S, Ninomiya K, Tomii D, Koseki K, Sato Y, Okuno T, Sato K, Koike H, Yahagi K, Komiyama K, Aoki J, Tanabe K. Impact of abdominal fat distribution, visceral fat, and subcutaneous fat on coronary plaque scores assessed by 320-row computed tomography coronary angiography. Atherosclerosis 287: 155–161, 2019. doi:10.1016/j.atherosclerosis.2019.06.910. 31295672

[6] Després J-P. Body fat distribution and risk of cardiovascular disease. Circulation 126: 1301–1313, 2012. doi:10.1161/CIRCULATIONAHA.111.067264. 22949540

[7] Tchkonia T, Thomou T, Zhu Y, Karagiannides I, Pothoulakis C, Jensen MD, Kirkland JL. Mechanisms and metabolic implications of regional differences among fat depots. Cell Metab 17: 644–656, 2013. doi:10.1016/j.cmet.2013.03.008. 23583168 PMC3942783

[8] Hruska P, Kucera J, Pekar M, Holéczy P, Mazur M, Buzga M, Kuruczova D, Lenart P, Fialova Kucerova J, Potesil D, Zdrahal Z, Bienertova-Vasku J. Proteomic signatures of human visceral and subcutaneous adipocytes. J Clin Endocrinol Metab 107: 755–775, 2022. doi:10.1210/clinem/dgab756. 34669916 PMC8851937

[9] Adamczyk P, Bužga M, Holéczy P, Švagera Z, Šmajstrla V, Zonča P, Pluskiewicz W. Bone mineral density and body composition after laparoscopic sleeve gastrectomy in men: a short-term longitudinal study. Int J Surg 23: 101–107, 2015. doi:10.1016/j.ijsu.2015.09.048. 26408948

[10] Adamczyk P, Bužga M, Holéczy P, Švagera Z, Zonča P, Sievänen H, Pluskiewicz W. Body size, bone mineral density, and body composition in obese women after laparoscopic sleeve gastrectomy: a 1-year longitudinal study. Horm Metab Res 47: 873–879, 2015. doi:10.1055/s-0035-1555758. 26134531

[11] Pluskiewicz W, Buzga M, Holeczy P, Šmajstrla V, Adamczyk P. A comment on “changes in bone mineral density in women following 1-year gastric bypass surgery” published by Casagrande DS, et al. Obes Surg 23: 1885, 2013. doi:10.1007/s11695-013-1018-8. 23921908

[12] Du Bois D, Du Bois EF. Clinical calorimetry: tenth paper a formula to estimate the approximate surface area if height and weight be known. Arch Intern Med (Chic) XVII: 863–871, 1916. doi:10.1001/archinte.1916.00080130010002.

[13] Aschner P. New IDF clinical practice recommendations for managing type 2 diabetes in primary care. Diabetes Res Clin Pract 132: 169–170, 2017. doi:10.1016/j.diabres.2017.09.002. 28962686

[14] Williams B, Mancia G, Spiering W, Agabiti Rosei E, Azizi M, Burnier M, Clement DL, Coca A, de Simone G, Dominiczak A, Kahan T, Mahfoud F, Redon J, Ruilope L, Zanchetti A, Kerins M, Kjeldsen SE, Kreutz R, Laurent S, Lip GYH, McManus R, Narkiewicz K, Ruschitzka F, Schmieder RE, Shlyakhto E, Tsioufis C, Aboyans V, Desormais I; ESC Scientific Document Group. 2018 ESC/ESH Guidelines for the management of arterial hypertension: the Task Force for the management of arterial hypertension of the European Society of Cardiology (ESC) and the European Society of Hypertension (ESH). Eur Heart J 39: 3021–3104, 2018 [Erratum in *Eur Heart J* 40: 475, 2019]. doi:10.1093/eurheartj/ehy339. 30165516

[15] Bužga M, Holéczy P, Švagera Z, Švorc P, Zavadilová V. Effects of sleeve gastrectomy on parameters of lipid and glucose metabolism in obese women—6 months after operation. Wideochir Inne Tech Maloinwazyjne 8: 22–28, 2013. doi:10.5114/wiitm.2011.31631. 23630550 PMC3627159

[16] Bužga M, Holéczy P, Švagera Z, Zonča P. Laparoscopic gastric plication and its effect on saccharide and lipid metabolism: a 12-month prospective study. Wideochir Inne Tech Maloinwazyjne 10: 398–405, 2015. doi:10.5114/wiitm.2015.54103. 26649086 PMC4653266

[17] Wiśniewski JR, Zougman A, Nagaraj N, Mann M. Universal sample preparation method for proteome analysis. Nat Methods 6: 359–362, 2009. doi:10.1038/nmeth.1322. 19377485

[18] Wiśniewski JR, Rakus D. Multi-enzyme digestion FASP and the ‘Total Protein Approach’-based absolute quantification of the Escherichia coli proteome. J Proteomics 109: 322–331, 2014. doi:10.1016/j.jprot.2014.07.012. 25063446

[19] Stejskal K, Potěšil D, Zdráhal Z. Suppression of peptide sample losses in autosampler vials. J Proteome Res 12: 3057–3062, 2013. doi:10.1021/pr400183v. 23590590

[20] Meier F, Geyer PE, Winter SV, Cox J, Mann M. BoxCar acquisition method enables single-shot proteomics at a depth of 10,000 proteins in 100 minutes. Nat Methods 15: 440–448, 2018. doi:10.1038/s41592-018-0003-5. 29735998

[21] Wichmann C, Meier F, Winter SV, Brunner A-D, Cox J, Mann M. MaxQuant.Live enables global targeting of more than 25,000 peptides. Mol Cell Proteomics 18: 982–994, 2019. doi:10.1074/mcp.TIR118.001131. 30755466 PMC6495250

[22] Cox J, Mann M. MaxQuant enables high peptide identification rates, individualized p.p.b.—range mass accuracies and proteome-wide protein quantification. Nat Biotechnol 26: 1367–1372, 2008. doi:10.1038/nbt.1511. 19029910

[23] Demichev V, Messner CB, Vernardis SI, Lilley KS, Ralser M. DIA-NN: neural networks and interference correction enable deep proteome coverage in high throughput. Nat Methods 17: 41–44, 2020. doi:10.1038/s41592-019-0638-x. 31768060 PMC6949130

[24] Perez-Riverol Y, Bai J, Bandla C, García-Seisdedos D, Hewapathirana S, Kamatchinathan S, Kundu DJ, Prakash A, Frericks-Zipper A, Eisenacher M, Walzer M, Wang S, Brazma A, Vizcaíno JA. The PRIDE database resources in 2022: a hub for mass spectrometry-based proteomics evidences. Nucleic Acids Res 50: D543–D552, 2022. doi:10.1093/nar/gkab1038. 34723319 PMC8728295

[25] R Development Core Team. R: A Language and Environment for Statistical Computing. VIenna, Austria: R Foundation for Statistical Computing, 2016. doi:10.1007/978-3-540-74686-7.

[26] Ritchie ME, Phipson B, Wu D, Hu Y, Law CW, Shi W, Smyth GK. Limma powers differential expression analyses for RNA-sequencing and microarray studies. Nucleic Acids Res 43: e47, 2015. doi:10.1093/nar/gkv007. 25605792 PMC4402510

[27] Bindea G, Mlecnik B, Hackl H, Charoentong P, Tosolini M, Kirilovsky A, Fridman W-H, Pagès F, Trajanoski Z, Galon J. ClueGO: a Cytoscape plug-in to decipher functionally grouped gene ontology and pathway annotation networks. Bioinformatics 25: 1091–1093, 2009. doi:10.1093/bioinformatics/btp101. 19237447 PMC2666812

[28] Jassal B, Matthews L, Viteri G, Gong C, Lorente P, Fabregat A, Sidiropoulos K, Cook J, Gillespie M, Haw R, Loney F, May B, Milacic M, Rothfels K, Sevilla C, Shamovsky V, Shorser S, Varusai T, Weiser J, Wu G, Stein L, Hermjakob H, D'Eustachio P. The reactome pathway knowledgebase. Nucleic Acids Res 48: D498–D503, 2020. doi:10.1093/nar/gkz1031. 31691815 PMC7145712

[29] Szklarczyk D, Gable AL, Lyon D, Junge A, Wyder S, Huerta-Cepas J, Simonovic M, Doncheva NT, Morris JH, Bork P, Jensen LJ, Mering C. V. STRING v11: protein-protein association networks with increased coverage, supporting functional discovery in genome-wide experimental datasets. Nucleic Acids Res 47: D607–D613, 2019. doi:10.1093/nar/gky1131. 30476243 PMC6323986

[30] Teufel F, Almagro Armenteros JJ, Johansen AR, Gíslason MH, Pihl SI, Tsirigos KD, Winther O, Brunak S, von Heijne G, Nielsen H. SignalP 6.0 predicts all five types of signal peptides using protein language models. Nat Biotechnol 40: 1023–1025, 2022. doi:10.1038/s41587-021-01156-3. 34980915 PMC9287161

[31] Langfelder P, Horvath S. Fast R functions for robust correlations and hierarchical clustering. J Stat Softw 46: i11, 2012. doi:10.18637/jss.v046.i11. 23050260 PMC3465711

[32] Langfelder P, Horvath S. WGCNA: an R package for weighted correlation network analysis. BMC Bioinformatics 9: 559, 2008. doi:10.1186/1471-2105-9-559. 19114008 PMC2631488

[33] Arderiu G, Mendieta G, Gallinat A, Lambert C, Díez-Caballero A, Ballesta C, Badimon L. Type 2 diabetes in obesity: a systems biology study on serum and adipose tissue proteomic profiles. Int J Mol Sci 24: 827, 2023. doi:10.3390/ijms24010827. 36614270 PMC9821208

[34] Insenser M, Montes-Nieto R, Vilarrasa N, Lecube A, Simó R, Vendrell J, Escobar-Morreale HF. A nontargeted proteomic approach to the study of visceral and subcutaneous adipose tissue in human obesity. Mol Cell Endocrinol 363: 10–19, 2012. doi:10.1016/j.mce.2012.07.001. 22796336

[35] Gómez-Serrano M, Camafeita E, García-Santos E, López JA, Rubio MA, Sánchez-Pernaute A, Torres A, Vázquez J, Peral B. Proteome-wide alterations on adipose tissue from obese patients as age-, diabetes- and gender-specific hallmarks. Sci Rep 6: 25756, 2016. doi:10.1038/srep25756. 27160966 PMC4861930

[36] Guzmán-Ruiz R, Tercero-Alcázar C, Rabanal-Ruiz Y, Díaz-Ruiz A, El Bekay R, Rangel-Zuñiga OA, Navarro-Ruiz MC, Molero L, Membrives A, Ruiz-Rabelo JF, Pandit A, López-Miranda J, Tinahones FJ, Malagón MM. Adipose tissue depot-specific intracellular and extracellular cues contributing to insulin resistance in obese individuals. FASEB J 34: 7520–7539, 2020. doi:10.1096/fj.201902703R. 32293066 PMC7384030

[37] Tang X, Li J, Zhao W-G, Sun H, Guo Z, Jing L, She Z, Yuan T, Liu S-N, Liu Q, Fu Y, Sun W. Comprehensive map and functional annotation of the mouse white adipose tissue proteome. PeerJ 7: e7352, 2019. doi:10.7717/peerj.7352. 31380149 PMC6661141

[38] Vogel MAA, Wang P, Bouwman FG, Hoebers N, Blaak EE, Renes J, Mariman EC, Goossens GH. A comparison between the abdominal and femoral adipose tissue proteome of overweight and obese women. Sci Rep 9: 4202, 2019. doi:10.1038/s41598-019-40992-x. 30862933 PMC6414508

[39] Ruiz-Ojeda FJ, Méndez-Gutiérrez A, Aguilera CM, Plaza-Díaz J. Extracellular matrix remodeling of adipose tissue in obesity and metabolic diseases. Int J Mol Sci 20: 4888, 2019. doi:10.3390/ijms20194888. 31581657 PMC6801592

[40] Khan T, Muise ES, Iyengar P, Wang ZV, Chandalia M, Abate N, Zhang BB, Bonaldo P, Chua S, Scherer PE. Metabolic dysregulation and adipose tissue fibrosis: role of collagen VI. Mol Cell Biol 29: 1575–1591, 2009. doi:10.1128/MCB.01300-08. 19114551 PMC2648231

[41] Pasarica M, Gowronska-Kozak B, Burk D, Remedios I, Hymel D, Gimble J, Ravussin E, Bray GA, Smith SR. Adipose tissue collagen VI in obesity. J Clin Endocrinol Metab 94: 5155–5162, 2009. doi:10.1210/jc.2009-0947. 19837927 PMC2819828

[42] Gesta S, Guntur K, Majumdar ID, Akella S, Vishnudas VK, Sarangarajan R, Narain NR. Reduced expression of collagen VI α 3 (COL6A3) confers resistance to inflammation-induced MCP1 expression in adipocytes. Obesity (Silver Spring) 24: 1695–1703, 2016. doi:10.1002/oby.21565. 27312141

[43] Lawler HM, Underkofler CM, Kern PA, Erickson C, Bredbeck B, Rasouli N. Adipose tissue hypoxia, inflammation, and fibrosis in obese insulin-sensitive and obese insulin-resistant subjects. J Clin Endocrinol Metab 101: 1422–1428, 2016. doi:10.1210/jc.2015-4125. 26871994 PMC4880157

[44] Spencer M, Yao-Borengasser A, Unal R, Rasouli N, Gurley CM, Zhu B, Peterson CA, Kern PA. Adipose tissue macrophages in insulin-resistant subjects are associated with collagen VI and fibrosis and demonstrate alternative activation. Am J Physiol Endocrinol Metab 299: E1016–E1027, 2010. doi:10.1152/ajpendo.00329.2010. 20841504 PMC3006260

[45] Liu Y, Aron-Wisnewsky J, Marcelin G, Genser L, Le Naour G, Torcivia A, Bauvois B, Bouchet S, Pelloux V, Sasso M, Miette V, Tordjman J, Clément K. Accumulation and changes in composition of collagens in subcutaneous adipose tissue after bariatric surgery. J Clin Endocrinol Metab 101: 293–304, 2016. doi:10.1210/jc.2015-3348. 26583585

[46] Nakazeki F, Nishiga M, Horie T, Nishi H, Nakashima Y, Baba O, Kuwabara Y, Nishino T, Nakao T, Ide Y, Koyama S, Kimura M, Tsuji S, Sowa N, Yoshida S, Conway SJ, Yanagita M, Kimura T, Ono K. Loss of periostin ameliorates adipose tissue inflammation and fibrosis in vivo. Sci Rep 8: 8553, 2018. doi:10.1038/s41598-018-27009-9. 29867212 PMC5986813

[47] Yang Y, Zhang Y, Zhou X, Chen D, Ouyang G, Liu Y, Cui D. Periostin deficiency attenuates lipopolysaccharide- and obesity-induced adipose tissue fibrosis. FEBS Lett 595: 2099–2112, 2021. doi:10.1002/1873-3468.14154. 34165806

[48] Chen X, Chen J, Xu D, Zhao S, Song H, Peng Y. Effects of osteoglycin (OGN) on treating senile osteoporosis by regulating MSCs. BMC Musculoskelet Disord 18: 423, 2017. doi:10.1186/s12891-017-1779-7. 29073887 PMC5658998

[49] Pessentheiner AR, Ducasa GM, Gordts PLSM. Proteoglycans in obesity-associated metabolic dysfunction and meta-inflammation. Front Immunol 11: 769, 2020. doi:10.3389/fimmu.2020.00769. 32508807 PMC7248225

[50] Sakashita H, Yamada S, Kinoshita M, Kajikawa T, Iwayama T, Murakami S. Mice lacking PLAP-1/asporin counteracts high fat diet-induced metabolic disorder and alveolar bone loss by controlling adipose tissue expansion. Sci Rep 11: 4970, 2021. doi:10.1038/s41598-021-84512-2. 33654143 PMC7925592

[51] Han CY, Kang I, Harten IA, Gebe JA, Chan CK, Omer M, Alonge KM, den Hartigh LJ, Gomes Kjerulf D, Goodspeed L, Subramanian S, Wang S, Kim F, Birk DE, Wight TN, Chait A. Adipocyte-derived versican and macrophage-derived biglycan control adipose tissue inflammation in obesity. Cell Rep 31: 107818, 2020. doi:10.1016/j.celrep.2020.107818. 32610121 PMC7384517

[52] Svensson L, Närlid I, Oldberg A. Fibromodulin and lumican bind to the same region on collagen type I fibrils. FEBS Lett 470: 178–182, 2000. doi:10.1016/s0014-5793(00)01314-4. 10734230

[53] Wolff G, Taranko AE, Meln I, Weinmann J, Sijmonsma T, Lerch S, Heide D, Billeter AT, Tews D, Krunic D, Fischer-Posovszky P, Müller-Stich BP, Herzig S, Grimm D, Heikenwälder M, Kao WW, Vegiopoulos A. Diet-dependent function of the extracellular matrix proteoglycan Lumican in obesity and glucose homeostasis. Mol Metab 19: 97–106, 2019. doi:10.1016/j.molmet.2018.10.007. 30409703 PMC6323191

[54] Kodama K, Horikoshi M, Toda K, Yamada S, Hara K, Irie J, Sirota M, Morgan AA, Chen R, Ohtsu H, Maeda S, Kadowaki T, Butte AJ. Expression-based genome-wide association study links the receptor CD44 in adipose tissue with type 2 diabetes. Proc Natl Acad Sci USA 109: 7049–7054, 2012. doi:10.1073/pnas.1114513109. 22499789 PMC3344989

[55] Petrus P, Fernandez TL, Kwon MM, Huang JL, Lei V, Safikhan NS, Karunakaran S, O'Shannessy DJ, Zheng X, Catrina S-B, Albone E, Laine J, Virtanen K, Clee SM, Kieffer TJ, Noll C, Carpentier AC, Johnson JD, Rydén M, Conway EM. Specific loss of adipocyte CD248 improves metabolic health via reduced white adipose tissue hypoxia, fibrosis and inflammation. EBioMedicine 44: 489–501, 2019. doi:10.1016/j.ebiom.2019.05.057. 31221584 PMC6606747

[56] Nicholls HT, Kowalski G, Kennedy DJ, Risis S, Zaffino LA, Watson N, Kanellakis P, Watt MJ, Bobik A, Bonen A, Febbraio M, Lancaster GI, Febbraio MA. Hematopoietic cell–restricted deletion of CD36 reduces high-fat diet–induced macrophage infiltration and improves insulin signaling in adipose tissue. Diabetes 60: 1100–1110, 2011. doi:10.2337/db10-1353. 21378177 PMC3064084

[57] Blaszczak AM, Wright VP, Anandani K, Liu J, Jalilvand A, Bergin S, Nicoloro SM, Czech MP, Lafuse W, Deng T, Bradley D, Hsueh WA. Loss of antigen presentation in adipose tissue macrophages or in adipocytes, but not both, improves glucose metabolism. J Immunol 202: 2451–2459, 2019. doi:10.4049/jimmunol.1801470. 30850480 PMC6822681

[58] Cho KW, Morris DL, DelProposto JL, Geletka L, Zamarron B, Martinez-Santibanez G, Meyer KA, Singer K, O'Rourke RW, Lumeng CN. An MHC class II dependent activation loop between adipose tissue macrophages and CD4+ T cells controls obesity-induced inflammation. Cell Rep 9: 605–617, 2014. doi:10.1016/j.celrep.2014.09.004. 25310975 PMC4252867

[59] Deng T, Lyon CJ, Minze LJ, Lin J, Zou J, Liu JZ, Ren Y, Yin Z, Hamilton DJ, Reardon PR, Sherman V, Wang HY, Phillips KJ, Webb P, Wong STC, Wang R-F, Hsueh WA. Class II major histocompatibility complex plays an essential role in obesity-induced adipose inflammation. Cell Metab 17: 411–422, 2013. doi:10.1016/j.cmet.2013.02.009. 23473035 PMC3619392

[60] Aziz MH, Cui K, Das M, Brown KE, Ardell CL, Febbraio M, Pluskota E, Han J, Wu H, Ballantyne CM, Smith JD, Cathcart MK, Yakubenko VP. The upregulation of integrin αDβ2 (CD11d/CD18) on inflammatory macrophages promotes macrophage retention in vascular lesions and development of atherosclerosis. J Immunol 198: 4855–4867, 2017. doi:10.4049/jimmunol.1602175. 28500072 PMC5553324

[61] Cui K, Ardell CL, Podolnikova NP, Yakubenko VP. Distinct migratory properties of M1, M2, and resident macrophages are regulated by αDβ2 and αMβ2 integrin-mediated adhesion. Front Immunol 9: 2650, 2018. doi:10.3389/fimmu.2018.02650. 30524429 PMC6262406

[62] Zheng C, Yang Q, Xu C, Shou P, Cao J, Jiang M, Chen Q, Cao G, Han Y, Li F, Cao W, Zhang L, Zhang L, Shi Y, Wang Y. CD11b regulates obesity-induced insulin resistance via limiting alternative activation and proliferation of adipose tissue macrophages. Proc Natl Acad Sci USA 112: E7239–E7248, 2015. doi:10.1073/pnas.1500396113. 26669445 PMC4702980

[63] Heinonen S, Jokinen R, Rissanen A, Pietiläinen KH. White adipose tissue mitochondrial metabolism in health and in obesity. Obes Rev 21: e12958, 2020. doi:10.1111/obr.12958. 31777187

[64] Chattopadhyay M, Guhathakurta I, Behera P, Ranjan KR, Khanna M, Mukhopadhyay S, Chakrabarti S. Mitochondrial bioenergetics is not impaired in nonobese subjects with type 2 diabetes mellitus. Metabolism 60: 1702–1710, 2011. doi:10.1016/j.metabol.2011.04.015. 21663924

[65] Gómez-Serrano M, Camafeita E, López JA, Rubio MA, Bretón I, García-Consuegra I, García-Santos E, Lago J, Sánchez-Pernaute A, Torres A, Vázquez J, Peral B. Differential proteomic and oxidative profiles unveil dysfunctional protein import to adipocyte mitochondria in obesity-associated aging and diabetes. Redox Biol 11: 415–428, 2017. doi:10.1016/j.redox.2016.12.013. 28064117 PMC5220168

[66] Schöttl T, Kappler L, Fromme T, Klingenspor M. Limited OXPHOS capacity in white adipocytes is a hallmark of obesity in laboratory mice irrespective of the glucose tolerance status. Mol Metab 4: 631–642, 2015. doi:10.1016/j.molmet.2015.07.001. 26413469 PMC4563017

[67] Wilson-Fritch L, Nicoloro S, Chouinard M, Lazar MA, Chui PC, Leszyk J, Straubhaar J, Czech MP, Corvera S. Mitochondrial remodeling in adipose tissue associated with obesity and treatment with rosiglitazone. J Clin Invest 114: 1281–1289, 2004. doi:10.1172/JCI21752. 15520860 PMC524228

[68] Fischer B, Schöttl T, Schempp C, Fromme T, Hauner H, Klingenspor M, Skurk T. Inverse relationship between body mass index and mitochondrial oxidative phosphorylation capacity in human subcutaneous adipocytes. Am J Physiol Endocrinol Metab 309: E380–E387, 2015. doi:10.1152/ajpendo.00524.2014. 26081284

[69] Kraunsøe R, Boushel R, Hansen CN, Schjerling P, Qvortrup K, Støckel M, Mikines KJ, Dela F. Mitochondrial respiration in subcutaneous and visceral adipose tissue from patients with morbid obesity. J Physiol 588: 2023–2032, 2010 [Erratum in *J Physiol* 588: 4055, 2010]. doi:10.1113/jphysiol.2009.184754. 20421291 PMC2911209

[70] Auguet T, Guiu-Jurado E, Berlanga A, Terra X, Martinez S, Porras JA, Ceausu A, Sabench F, Hernandez M, Aguilar C, Sirvent JJ, Del Castillo D, Richart C. Downregulation of lipogenesis and fatty acid oxidation in the subcutaneous adipose tissue of morbidly obese women. Obesity (Silver Spring) 22: 2032–2038, 2014. doi:10.1002/oby.20809. 24931172

[71] Arner P, Bernard S, Salehpour M, Possnert G, Liebl J, Steier P, Buchholz BA, Eriksson M, Arner E, Hauner H, Skurk T, Rydén M, Frayn KN, Spalding KL. Dynamics of human adipose lipid turnover in health and metabolic disease. Nature 478: 110–113, 2011. doi:10.1038/nature10426. 21947005 PMC3773935

[72] Arner P, Rydén M. Human white adipose tissue: a highly dynamic metabolic organ. J Intern Med 291: 611–621, 2022. doi:10.1111/joim.13435. 34914848

[73] Lemmer IL, Willemsen N, Hilal N, Bartelt A. A guide to understanding endoplasmic reticulum stress in metabolic disorders. Mol Metab 47: 101169, 2021. doi:10.1016/j.molmet.2021.101169. 33484951 PMC7887651

[74] Slimen IB, Najar T, Ghram A, Dabbebi H, Ben Mrad M, Abdrabbah M. Reactive oxyen species, heat stress and oxidative-induced mitochondrial damage. A review. Int J Hyperthermia 30: 513–523, 2014. doi:10.3109/02656736.2014.971446. 25354680

[75] Yin L, Huang D, Liu X, Wang Y, Liu J, Liu F, Yu B. Omentin-1 effects on mesenchymal stem cells: proliferation, apoptosis, and angiogenesis in vitro. Stem Cell Res Ther 8: 224, 2017. doi:10.1186/s13287-017-0676-1. 29017592 PMC5633887

[76] Liu F, Fang S, Liu X, Li J, Wang X, Cui J, Chen T, Li Z, Yang F, Tian J, Li H, Yin L, Yu B. Omentin-1 protects against high glucose-induced endothelial dysfunction via the AMPK/PPARδ signaling pathway. Biochem Pharmacol 174: 113830, 2020. doi:10.1016/j.bcp.2020.113830. 32001235

[77] Francisco V, Ruiz-Fernández C, Pino J, Mera A, González-Gay MA, Gómez R, Lago F, Mobasheri A, Gualillo O. Adipokines: linking metabolic syndrome, the immune system, and arthritic diseases. Biochem Pharmacol 165: 196–206, 2019. doi:10.1016/j.bcp.2019.03.030. 30910694

[78] Zhao S, Kusminski CM, Scherer PE. Adiponectin, leptin and cardiovascular disorders. Circ Res 128: 136–149, 2021. doi:10.1161/CIRCRESAHA.120.314458. 33411633 PMC7799441

[79] Neeland IJ, Ayers CR, Rohatgi AK, Turer AT, Berry JD, Das SR, Vega GL, Khera A, McGuire DK, Grundy SM, de Lemos JA. Associations of visceral and abdominal subcutaneous adipose tissue with markers of cardiac and metabolic risk in obese adults. Obesity (Silver Spring) 21: E439–E447, 2013. doi:10.1002/oby.20135. 23687099 PMC3751977

[80] Samaras K, Botelho NK, Chisholm DJ, Lord RV. Subcutaneous and visceral adipose tissue gene expression of serum adipokines that predict type 2 diabetes. Obesity (Silver Spring) 18: 884–889, 2010. doi:10.1038/oby.2009.443. 20019678

[81] Mejhert N, Wilfling F, Esteve D, Galitzky J, Pellegrinelli V, Kolditz C-I, Viguerie N, Tordjman J, Näslund E, Trayhurn P, Lacasa D, Dahlman I, Stich V, Lång P, Langin D, Bouloumié A, Clément K, Rydén M. Semaphorin 3C is a novel adipokine linked to extracellular matrix composition. Diabetologia 56: 1792–1801, 2013. doi:10.1007/s00125-013-2931-z. 23666167

[82] Nam JS, Ahn CW, Park HJ, Kim YS. Semaphorin 3 C is a novel adipokine representing exercise-induced improvements of metabolism in metabolically healthy obese young males. Sci Rep 10: 10005, 2020. doi:10.1038/s41598-020-67004-7. 32561824 PMC7305109

[83] Lu Y-C, Chang C-C, Wang C-P, Hung W-C, Tsai I-T, Tang W-H, Wu C-C, Wei C-T, Chung F-M, Lee Y-J, Hsu C-C. Circulating fatty acid-binding protein 1 (FABP1) and nonalcoholic fatty liver disease in patients with type 2 diabetes mellitus. Int J Med Sci 17: 182–190, 2020. doi:10.7150/ijms.40417. 32038102 PMC6990891

[84] Tsai I-T, Wu C-C, Hung W-C, Lee T-L, Hsuan C-F, Wei C-T, Lu Y-C, Yu T-H, Chung F-M, Lee Y-J, Wang C-P. FABP1 and FABP2 as markers of diabetic nephropathy. Int J Med Sci 17: 2338–2345, 2020. doi:10.7150/ijms.49078. 32922199 PMC7484639

